# Supplementary material for: Acidic media enables oxygen-tolerant electrosynthesis of multicarbon products from simulated flue gas
Source: Nat Commun. 2024 Feb 9;15:1218. doi: 10.1038/s41467-024-45527-1 (PMC10858036; doi:10.1038/s41467-024-45527-1)
Supplement: Supplementary file 1 — Supplementary Information [file 41467_2024_45527_MOESM1_ESM.pdf]

Supplementary Information

Acidic media enables oxygen-tolerant electrosynthesis of multicarbon products from simulated flue gas

Meng Wang[1,2,3,5], Bingqing Wang[1,5*], Jiguang Zhang[1,2,5], Shibo Xi[4], Ning Ling[1], Ziyu Mi[4], Qin Yang[1], Mingsheng Zhang[2], Wan Ru Leow[4], Jia Zhang[3] and Yanwei Lum[1,2]*

[1]Department of Chemical and Biomolecular Engineering, National University of Singapore, Singapore, 117585 Republic of Singapore

[2]Institute of Materials Research and Engineering, Agency for Science, Technology and Research (A*STAR), 2 Fusionopolis Way, Innovis #08-03, Singapore, 138634 Republic of Singapore

[3]Institute of High Performance Computing, Agency for Science, Technology, and Research (A*STAR), 1 Fusionopolis Way, #16-16 Connexis, Singapore, 138632 Republic of Singapore

[4]Institute of Sustainability for Chemicals, Energy and Environment (ISCE2), Agency for Science, Technology and Research (A*STAR), 1 Pesek Road, Singapore, 627833 Republic of Singapore

[5]These authors contributed equally to this work.

*Corresponding author: wangbq@nus.edu.sg

*Corresponding author: lumyw@nus.edu.sg

This file includes:

Figure S1- S43

Table S1-S41

Supplementary References

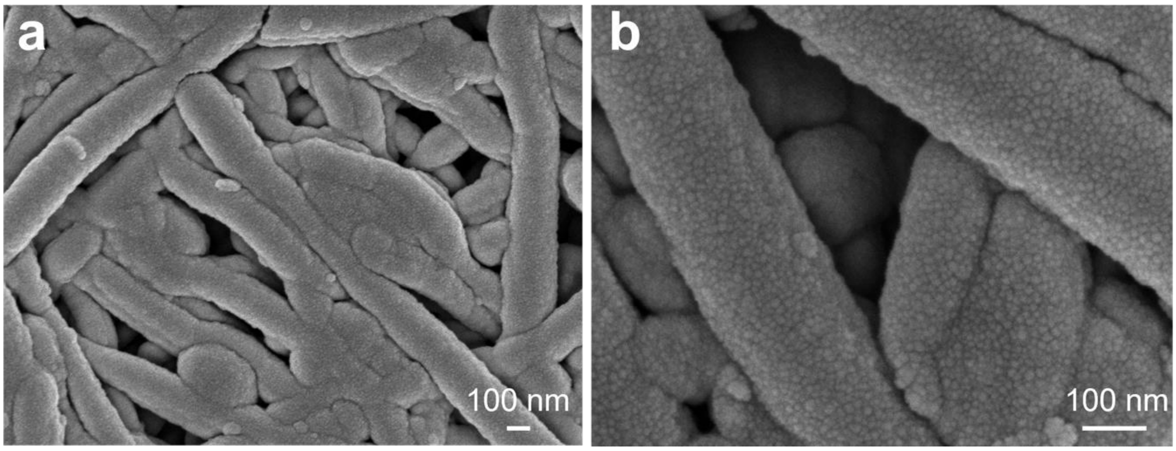

Figure S1. Scanning electron microscopy (SEM) images of Cu PTFE under: (a) low and (b) high degree of magnification. The porous PTFE substrate has a pore size of 0.45 μm and consists of a web-like morphology with individual fibres coated conformally with the Cu catalyst.

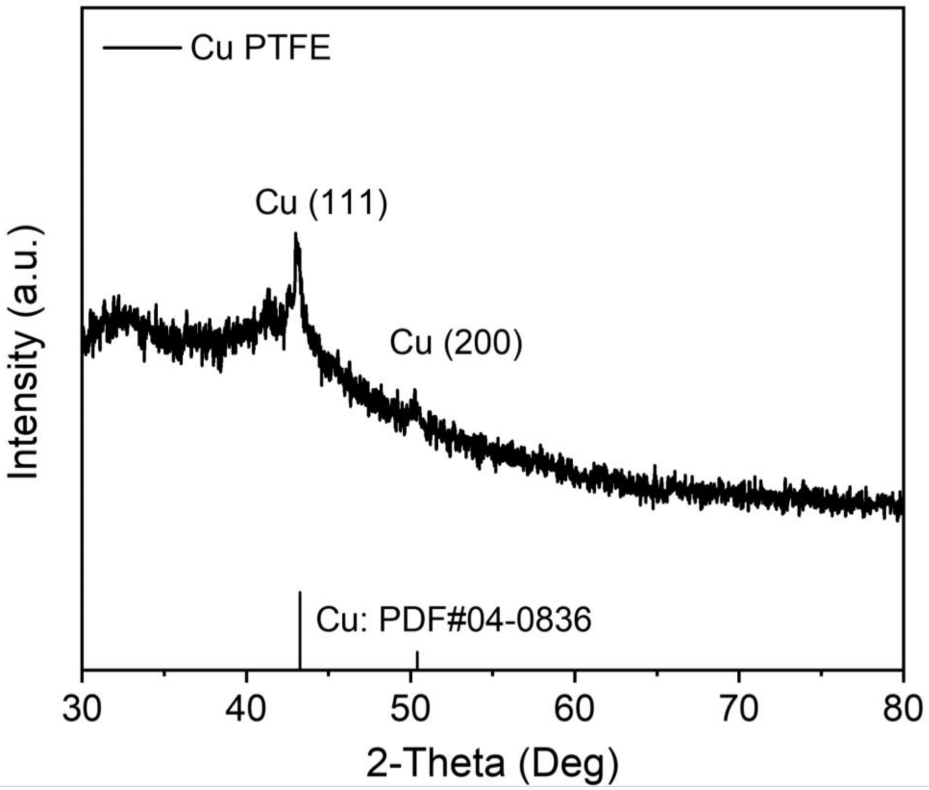

Figure S2. XRD patterns of Cu PTFE, where we observe the dominant peak to be Cu (111). Cu: PDF#04-0836 is shown as a reference.

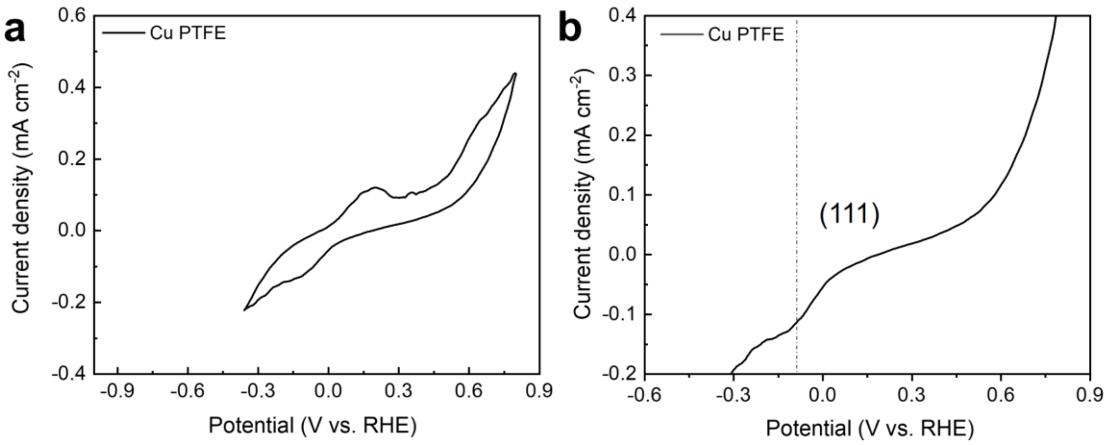

Figure S3. (a) Cyclic voltammogram recorded with Cu PTFE in 0.1 M $HClO_4$ aqueous solution containing 1 mM $Pb(ClO_4)_2$. (b) is the zoomed-in view of the cathodic peaks, which correspond to the deposition of Pb onto the Cu (111) facet. The cyclic voltammogram was recorded at a scan rate of 10 mV s^{-1}.

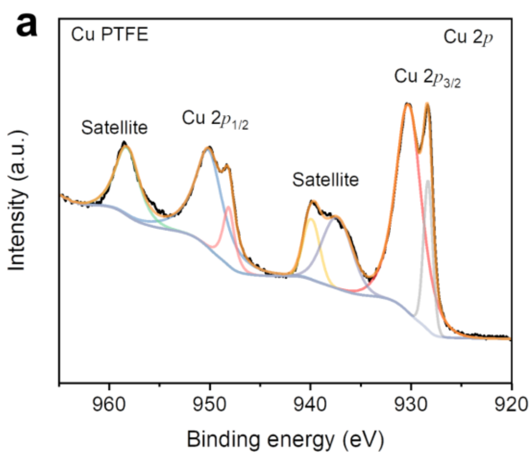

Figure S4. (a) Narrow scan X-ray photoelectron spectroscopy (XPS) spectrums of Cu 2*p* and (b) survey scan of Cu PTFE.

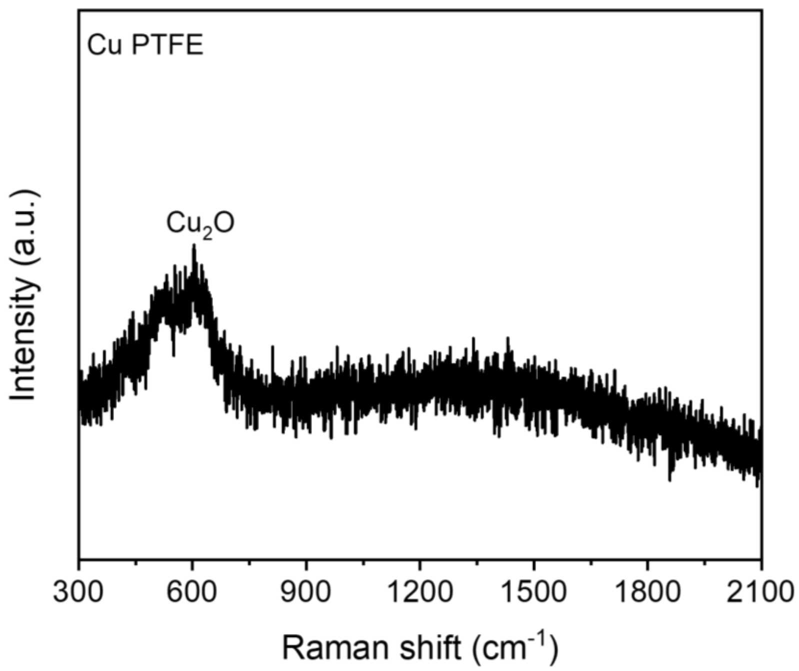

Figure S5. Raman spectroscopy data of Cu PTFE. A Cu_2O peak is observed, which forms due to oxidation of the surface upon exposure to ambient air.

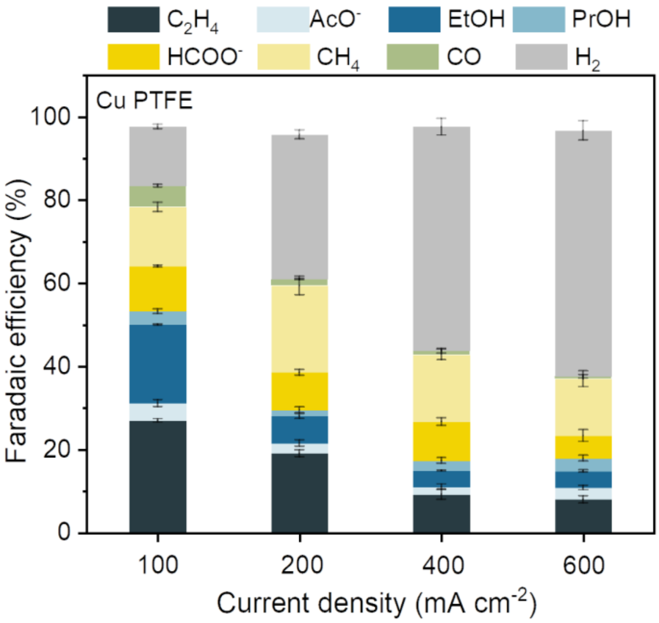

Figure S6. FE results of Cu PTFE for CO_2R in acidic electrolyte at cathodic current densities of 100, 200, 400 and 600 mA cm^{-2} (this data is also shown as Figure 3a in the main text). All the error bars represent standard deviation based on three independent samples.

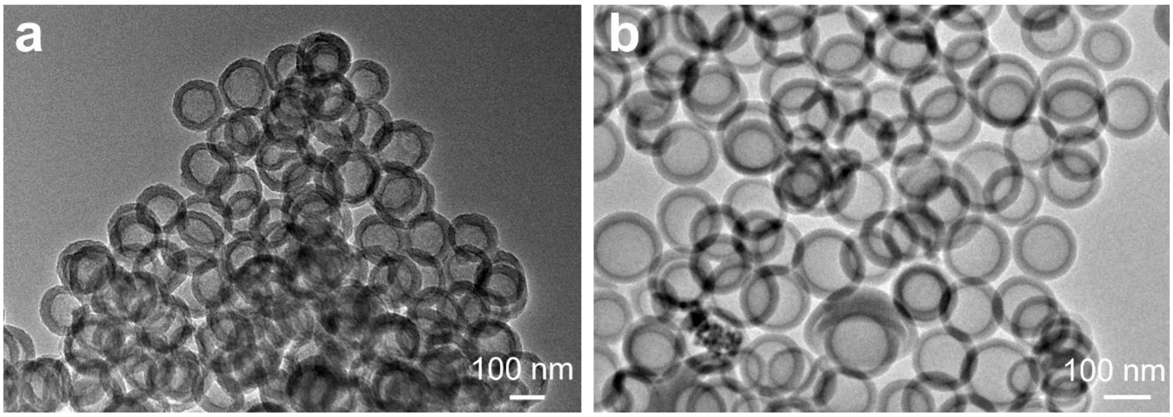

Figure S7. TEM images of the Ni-N_4 catalyst under: (a) low and (b) high degree of magnification. These consist of Ni single atoms homogenously dispersed onto a carbon support.

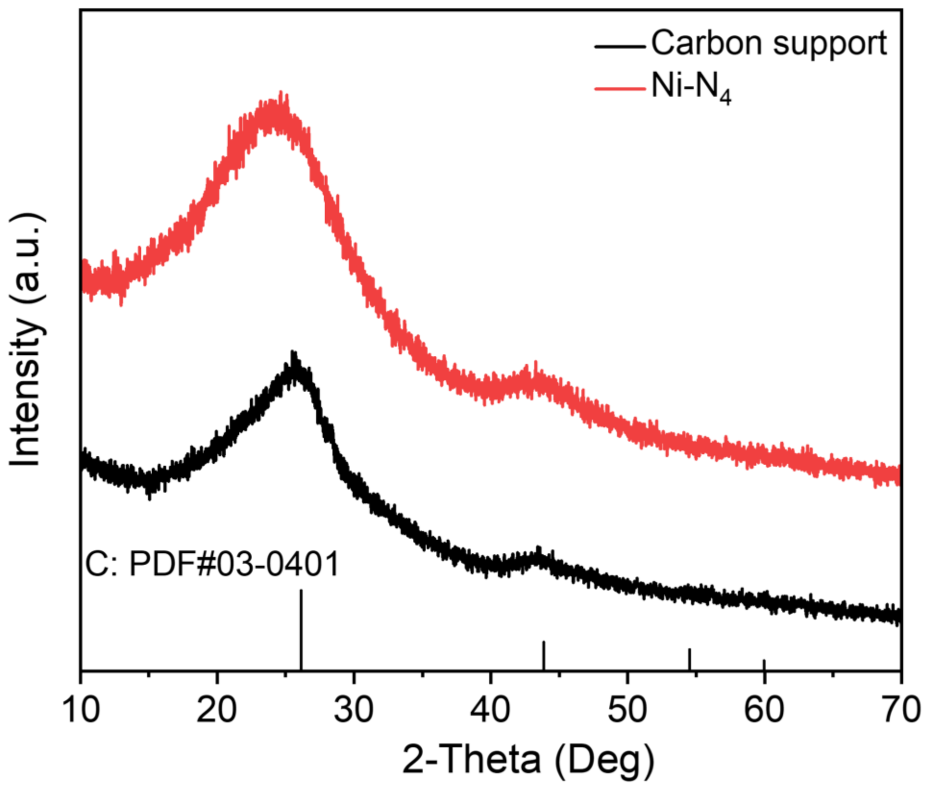

Figure S8. XRD patterns of the bare carbon support and the Ni-N_4 catalyst. C: PDF#03-0401 is shown as a reference.

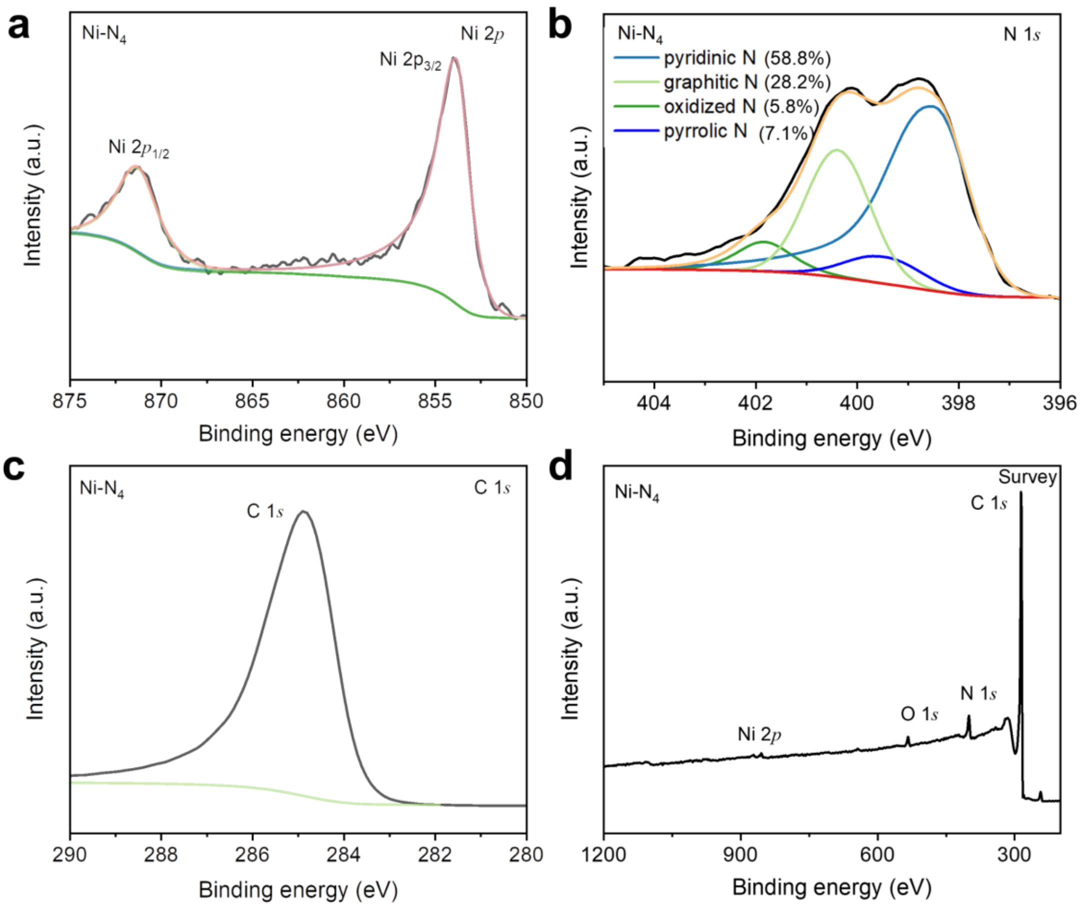

Figure S9. Narrow scan X-ray photoelectron spectroscopy (XPS) spectrums of (a) Ni 2*p*, (b) N 1*s*, (c) C 1*s* and (d) survey scan for the Ni-N_4 catalyst.

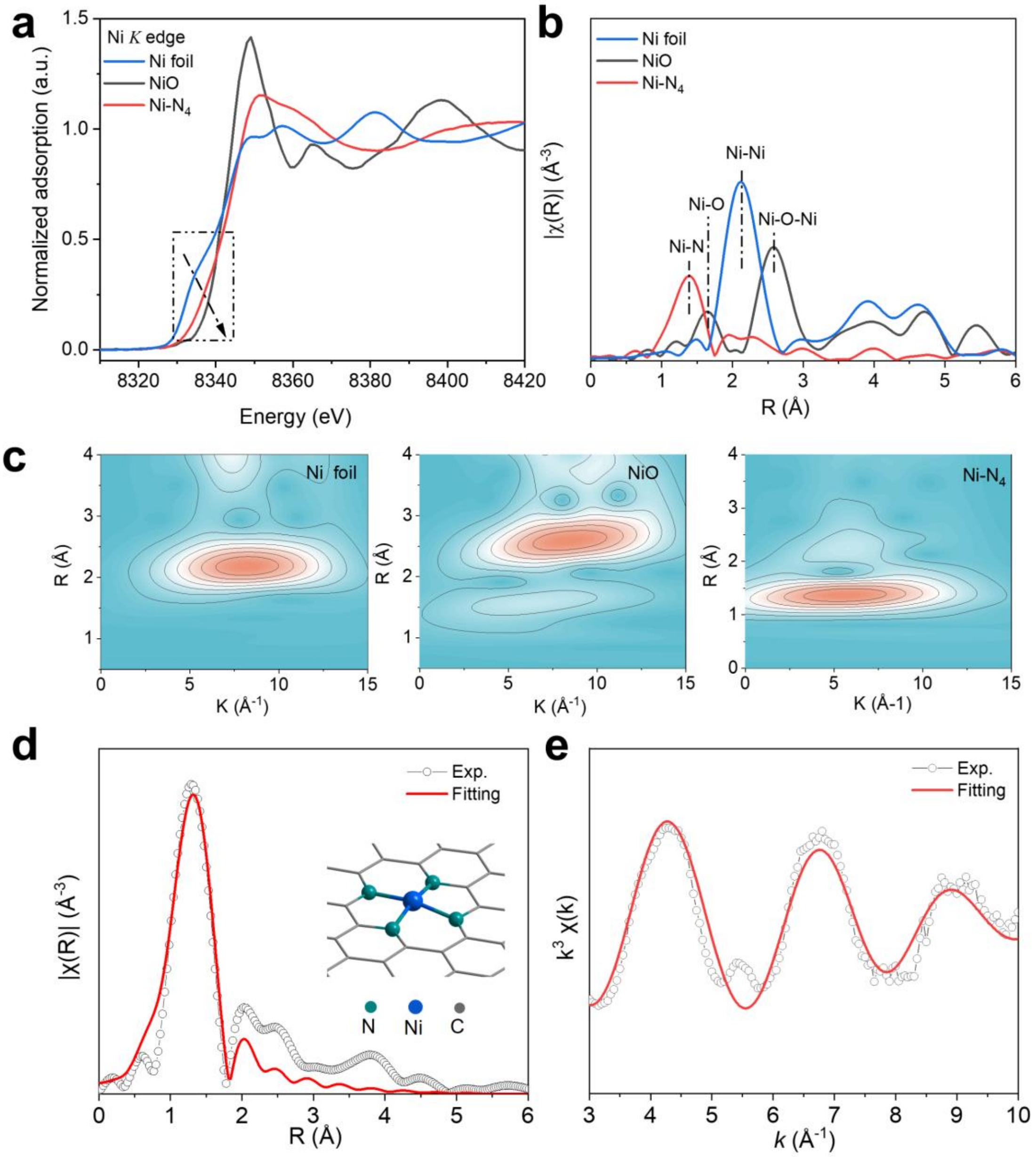

Figure S10. (a) Ni *K*-edge XANES spectra and (b) Ni *K*-edge Fourier-transformed (FT) k^2-weighted χ(k) functions of Ni-N_4. NiO and Ni foil were used as references. (c) Ni *K* edge wavelet transform analysis of the Ni-N_4 catalyst. EXAFS fitting results of the Ni K edge of the Ni-N_4 in (d) R space and (e) k space.

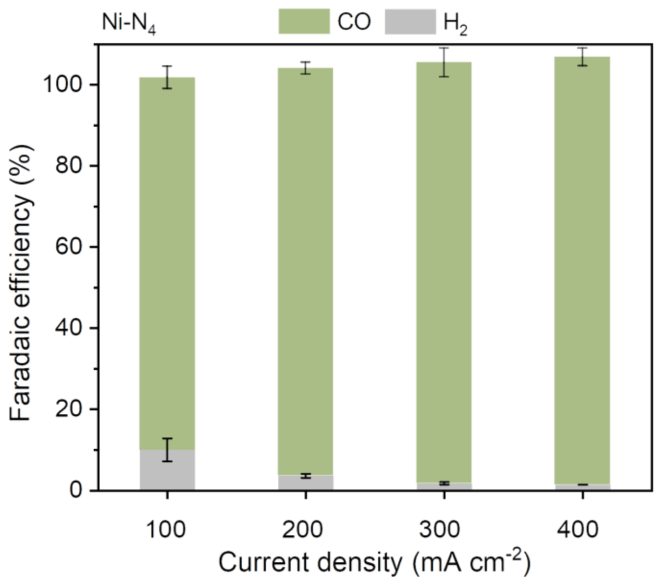

Figure S11. CO_2R results for the Ni-N_4 catalysts in acidic electrolyte at cathodic current densities of 100, 200, 300 and 400 mA cm^{-2}. All the error bars represent standard deviation based on three independent samples.

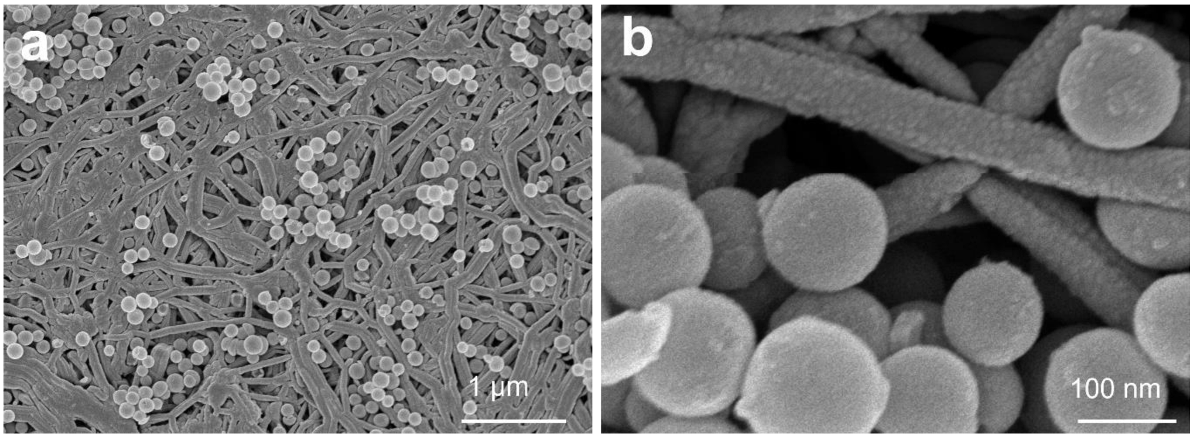

Figure S12. SEM images of Ni-N_4 loading on Cu PTFE under: (a) low and (b) high degree of magnification.

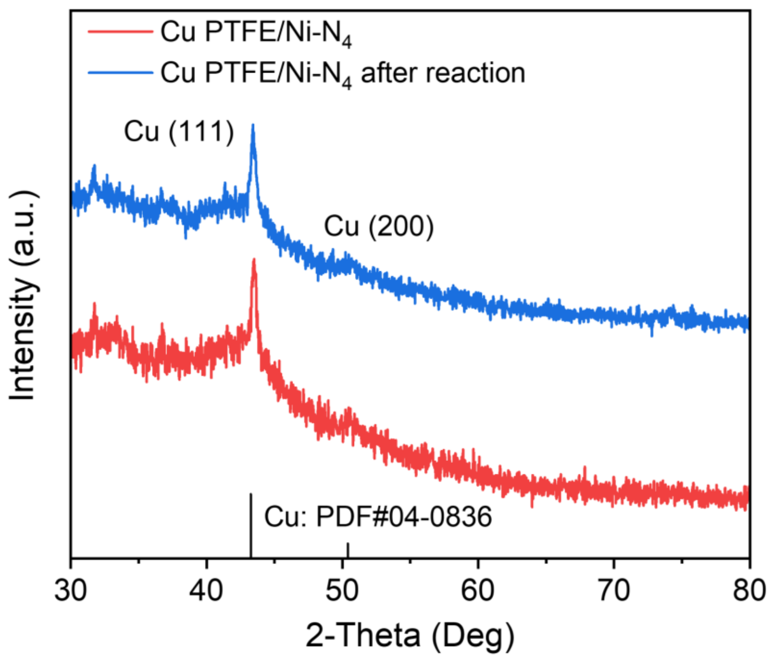

Figure S13. XRD patterns of the Cu PTFE/Ni-N_4 catalyst before and after CO_2R experiments.

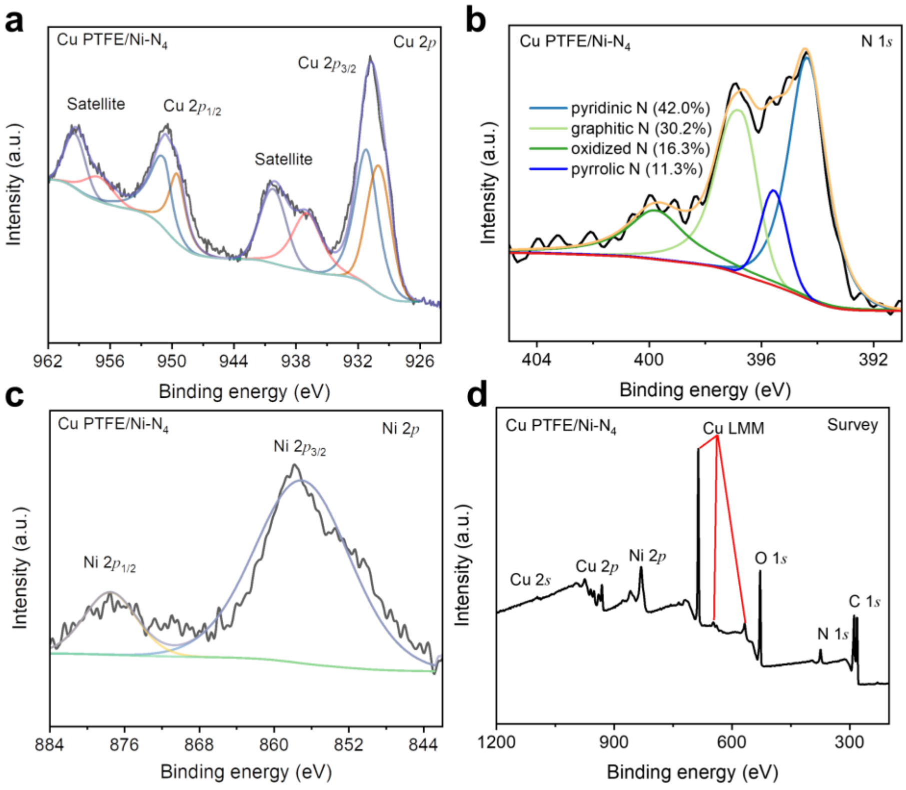

Figure S14. Narrow scan X-ray photoelectron spectroscopy (XPS) spectrums of (a) Cu 2*p*, (b) N 1*s*, (c) Ni 2*p* and (d) survey scan for Cu PTFE/Ni-N_4.

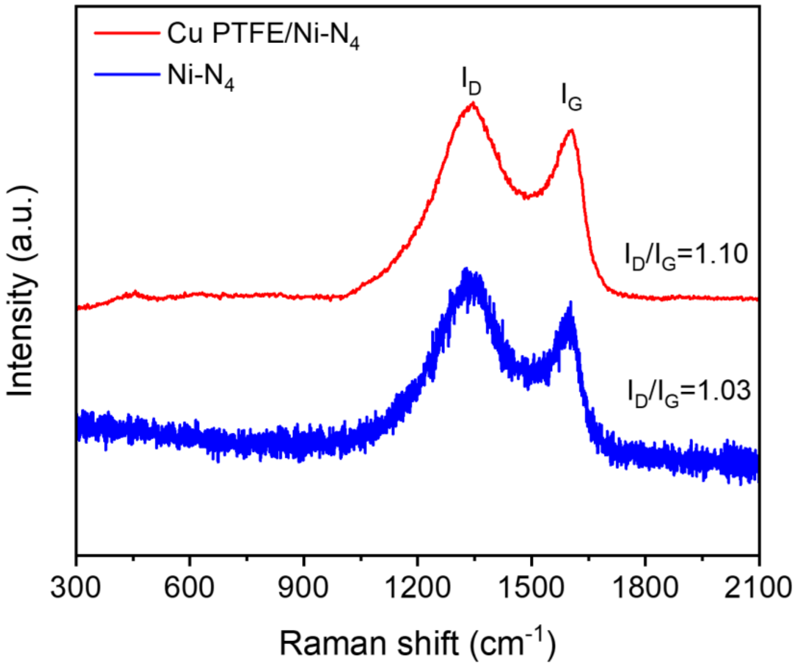

Figure S15. Raman spectroscopy data of Ni-N_4 and Cu PTFE/Ni-N_4. No significant differences in the spectra are observed, which indicates that the coating process does not directly alter the properties of Ni-N_4.

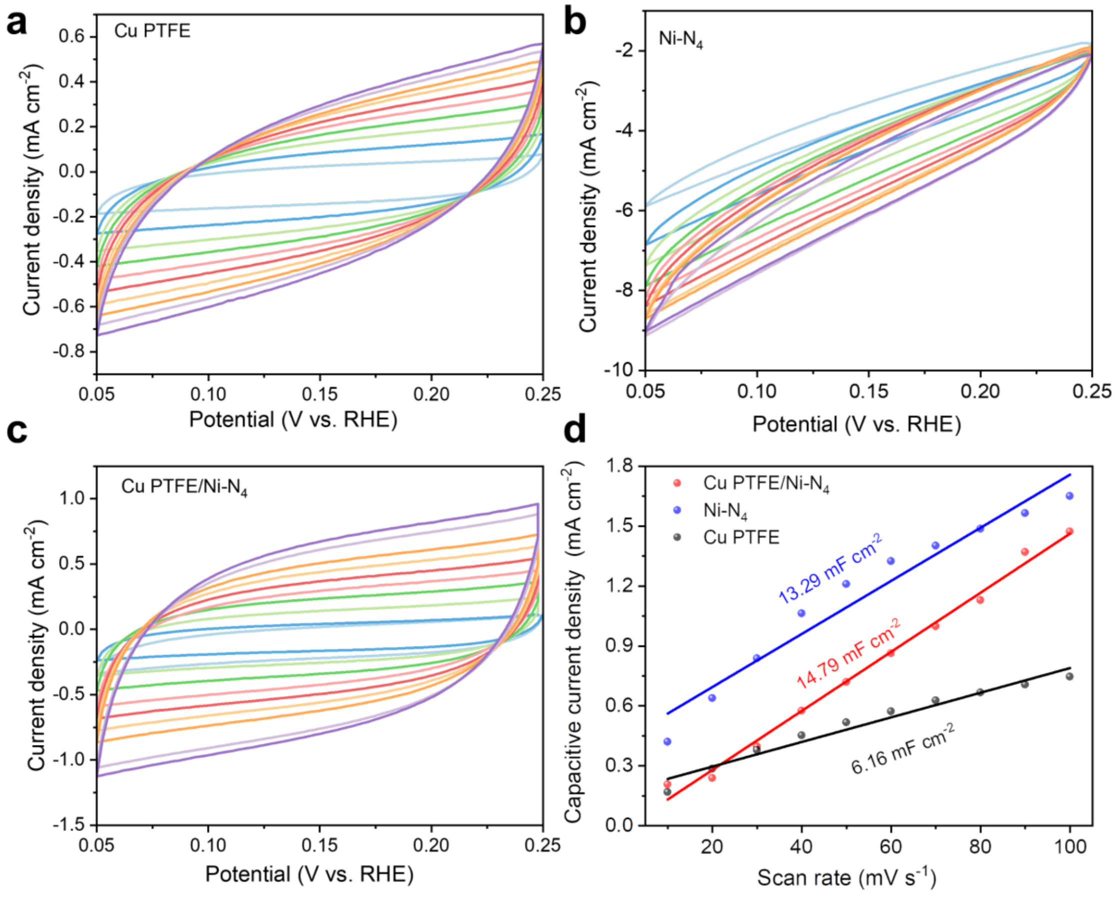

Figure S16. Cyclic voltammograms for (a) Cu PTFE, (b) $Ni\text{-}N_4$, and (c) Cu PTFE/$Ni\text{-}N_4$ at different scan rates from 10 to 100 mV s^{-1} in a potential range where Faradaic reactions do not occur. (d) shows the corresponding plots of the capacitive current vs scan rate, with the calculated capacitance (slope) shown for each case.

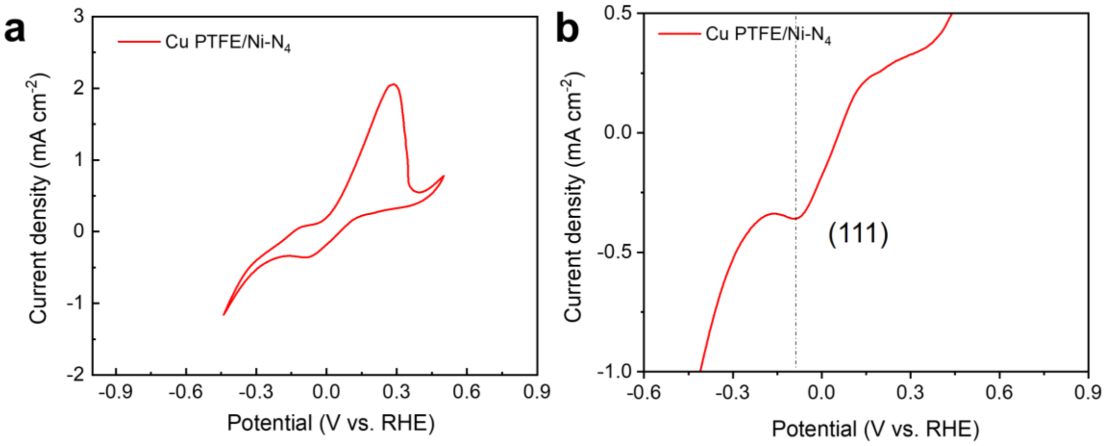

Figure S17. (a) Cyclic voltammogram recorded on Cu PTFE/Ni-N_4 in 0.1 M $HClO_4$ aqueous solution with 1 mM $Pb(ClO_4)_2$. (b) is the zoomed-in view of the cathodic peaks of the cyclic voltammograms, showing the deposition of Pb on Cu (111) facet. The curves were recorded at a scan rate of 10 mV s^{-1}.

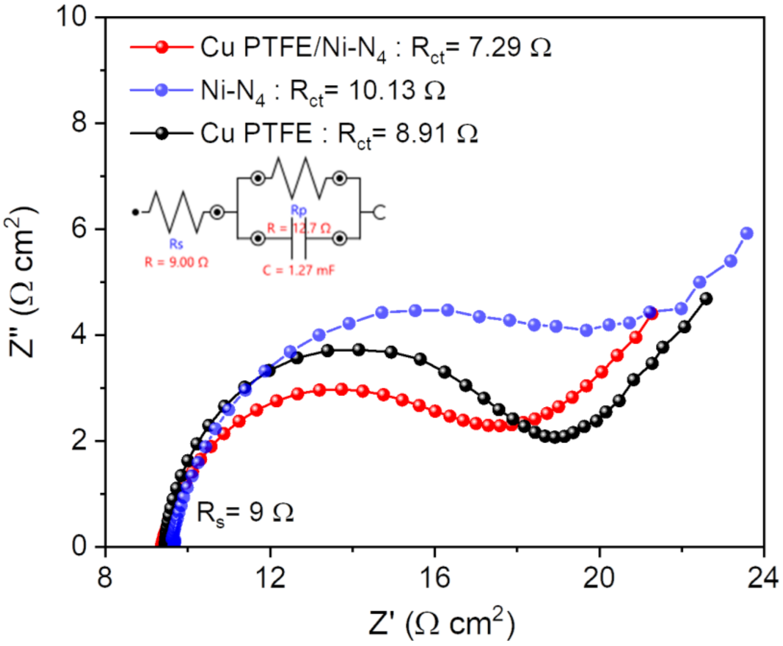

Figure S18. Nyquist plots for Cu PTFE, Ni-N4 and Cu PTFE/Ni-N_4. Shown on the plot are the solution resistance (R_s) and the charge transfer resistance (R_{ct}).

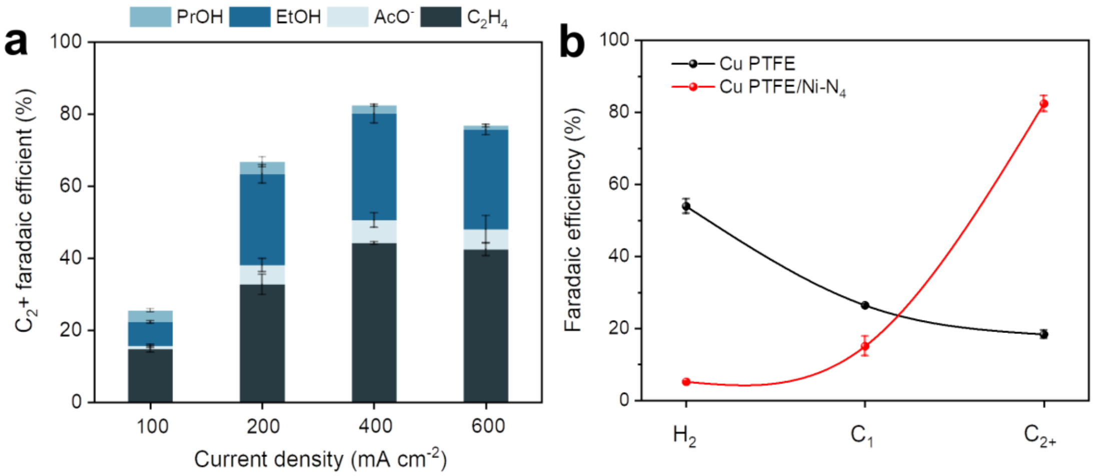

Figure S19. (a) FEs of C_{2+} products on Cu PTFE/Ni-N_4 under different current densities in a flow cell. (b) FE comparison between Cu PTFE and Cu PTFE/Ni-N_4 at 400 mA cm^{-2}. All the error bars represent standard deviation based on three independent samples.

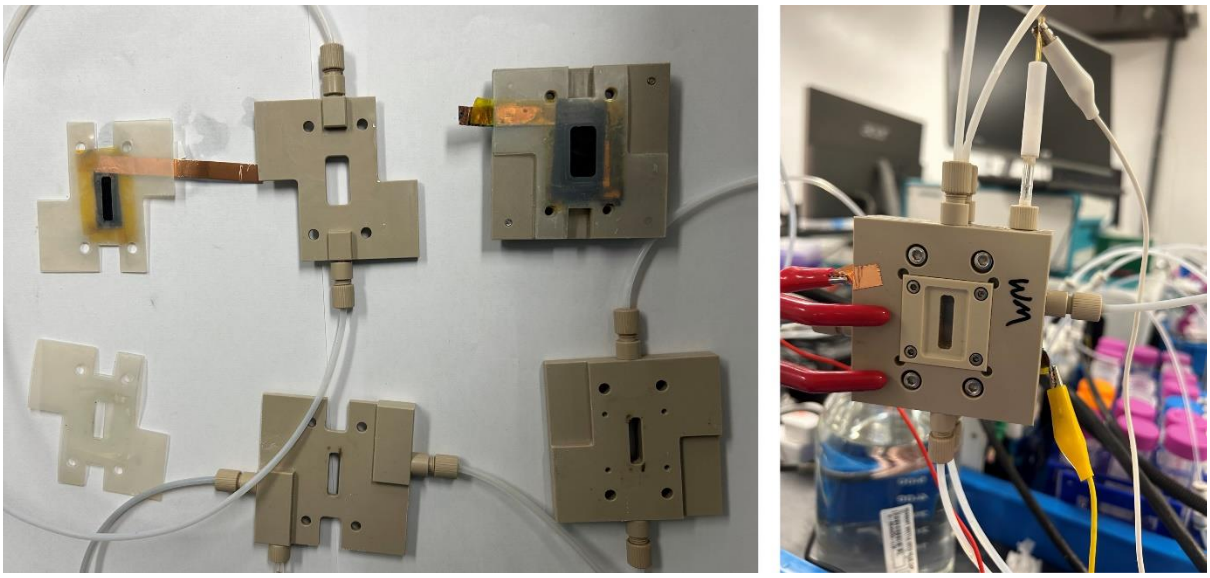

Figure S20. Custom-built electrochemical flow cell used for CO_2R test.

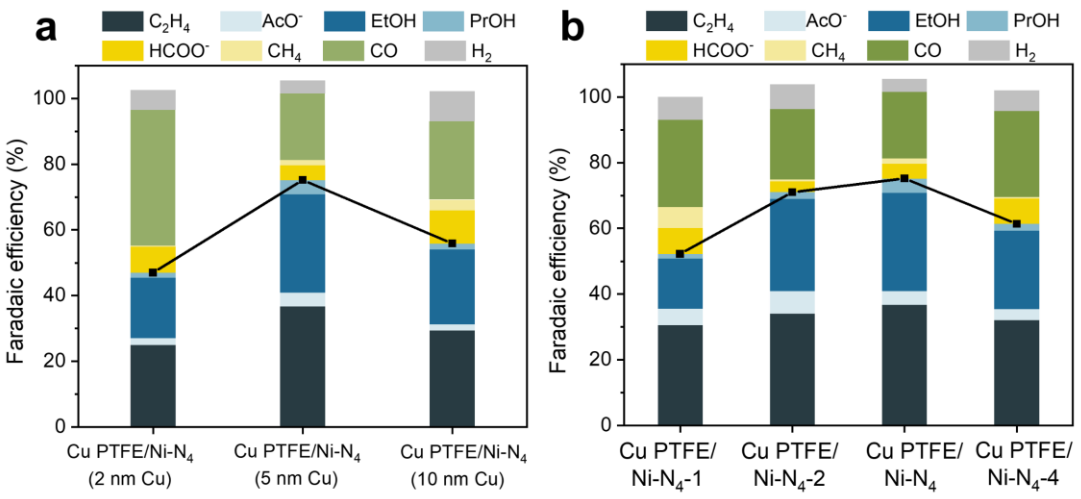

Figure S21. (a) FE results for Cu PTFE/Ni-N_4 with different thickness (2, 5 and 10 nm) of sputtered Cu. Electrolyte used was 0.05 M H_2SO_4 + 0.5 M K_2SO_4 at a cathodic current density of 200 mA cm^{-2}. (b) FE results for Cu PTFE/Ni-N_4-1, Cu PTFE/Ni-N_4-2, Cu PTFE/Ni-N_4, and Cu PTFE/Ni-N_4-4 at a cathodic current density of 200 mA cm^{-2} in 0.05 M H_2SO_4 + 0.5 M K_2SO_4 electrolyte. All the error bars represent standard deviation based on three independent samples.

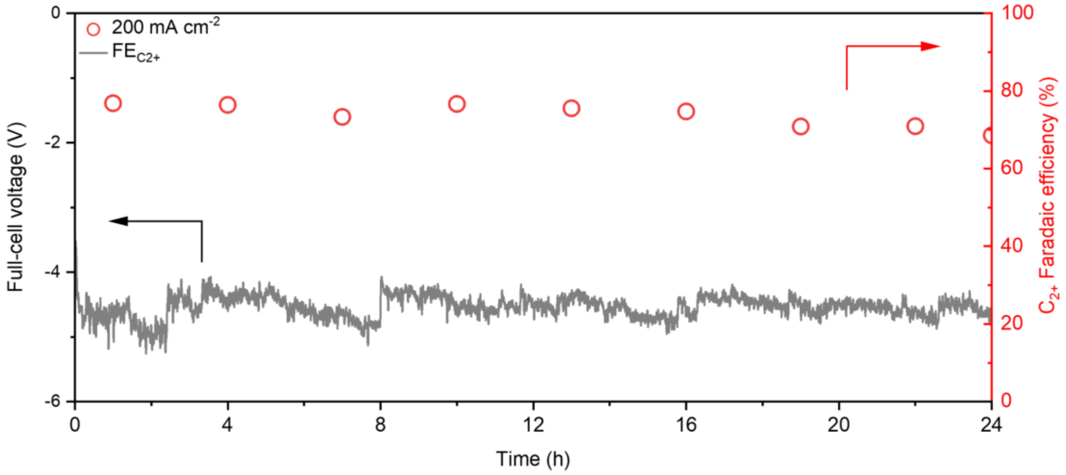

Figure S22. Full-cell voltage and FE towards C_{2+} products measured during 24 h of continuous operation at a current density of 200 mA cm^{-2}.

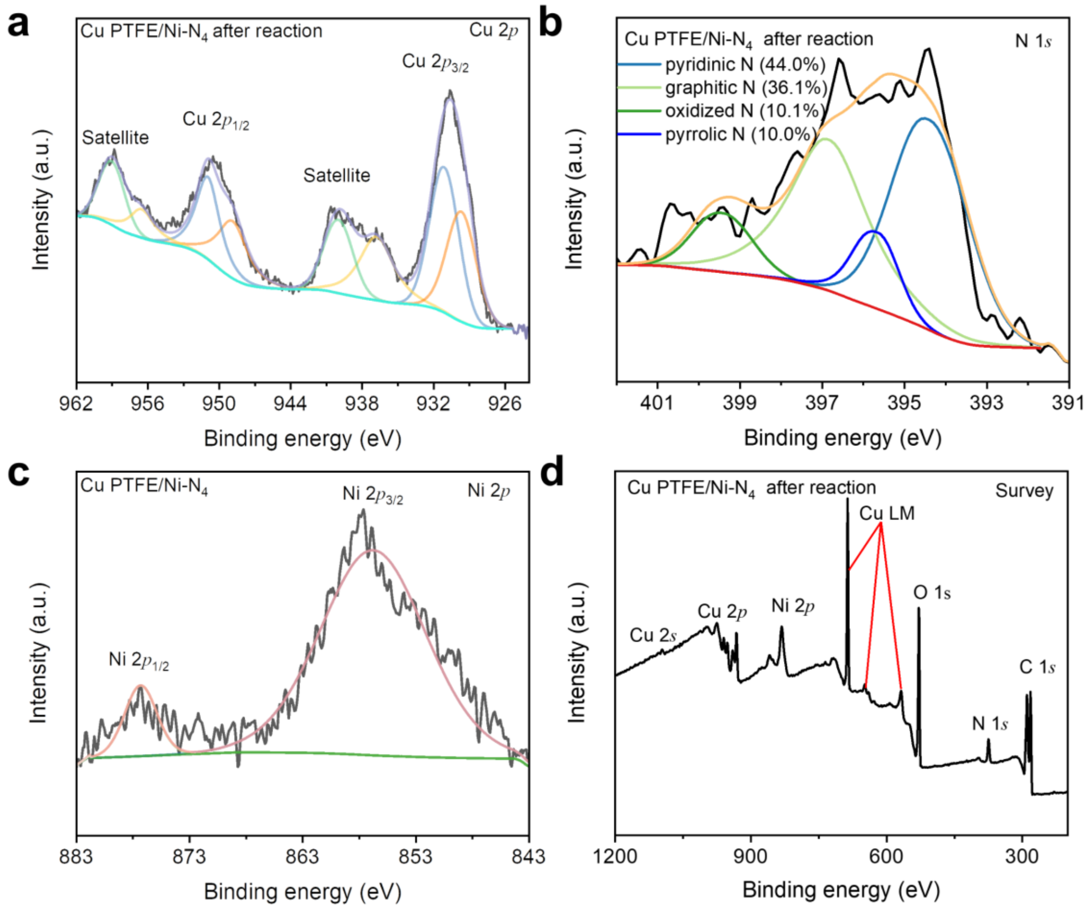

Figure S23. Narrow scan X-ray photoelectron spectroscopy (XPS) spectrums of Cu 2*p* peak (a), N 1*s* (b), Ni 2*p* (c) and survey scan XPS spectrums (d) for Cu PTFE/Ni-N_4 after CO_2R.

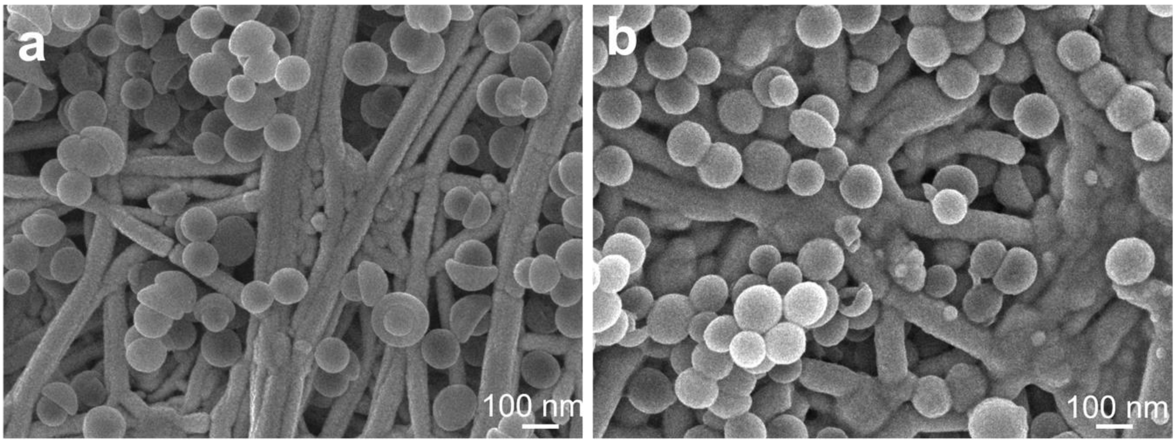

Figure S24. (a) and (b) are SEM images of Cu PTFE/Ni-N_4 after CO_2R experiments.

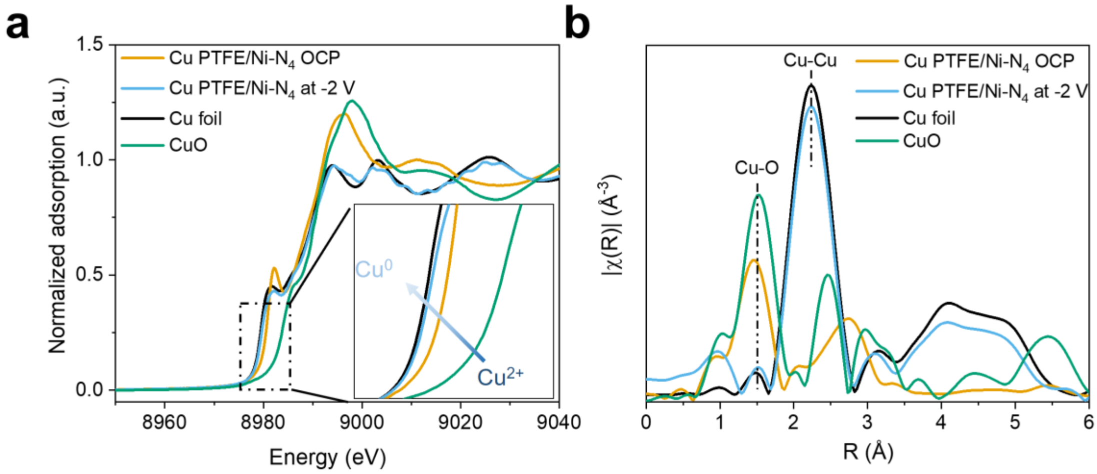

Figure S25. (a) *In-situ* Cu k-edge XANES spectra of Cu PTFE/Ni-N_4 (OCP), Cu PTFE/Ni-N_4 at -2 V versus RHE. (b) Fourier transform of k^2-weighted χ function in R space of Cu PTFE/Ni-N_4 plus Cu foil and CuO as reference.

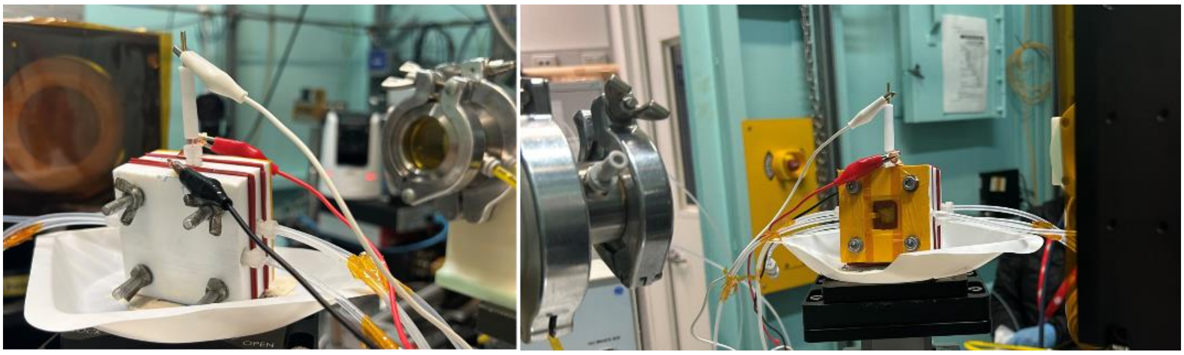

Figure S26. Custom-built electrochemical cell used for *in-situ* X-ray absorption spectroscopy (XAS) experiments.

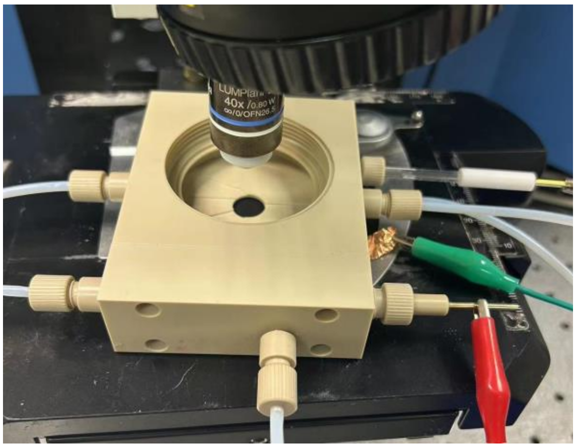

Figure S27. Custom-built electrochemical flow cell for *in-situ* Raman spectroscopy experiments.

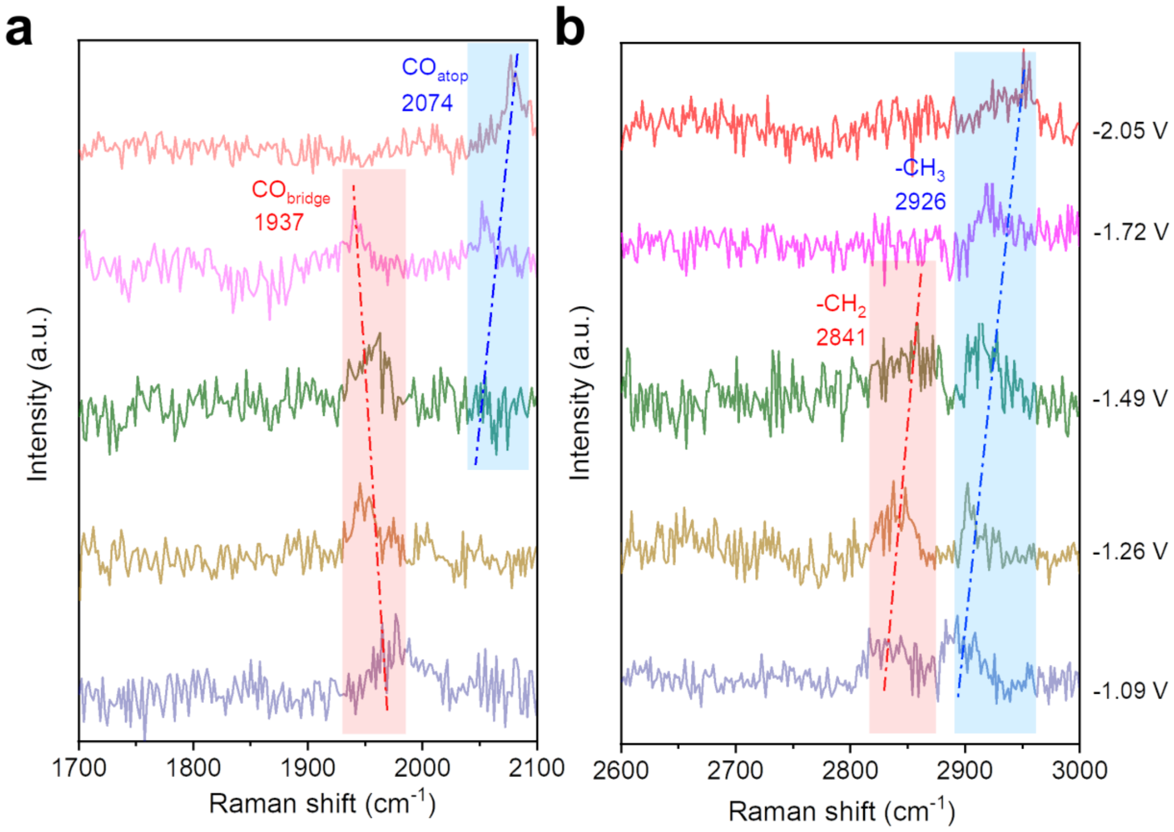

Figure S28. *In-situ* Raman spectroscopy at (a) 1,600-2,100 cm^{-1} and (b) 2,600-3,000 cm^{-1} of the Cu PTFE/Ni-N_4 catalyst measured in 0.05 M H_2SO_4 + 0.5 M K_2SO_4 (acidic) electrolyte.

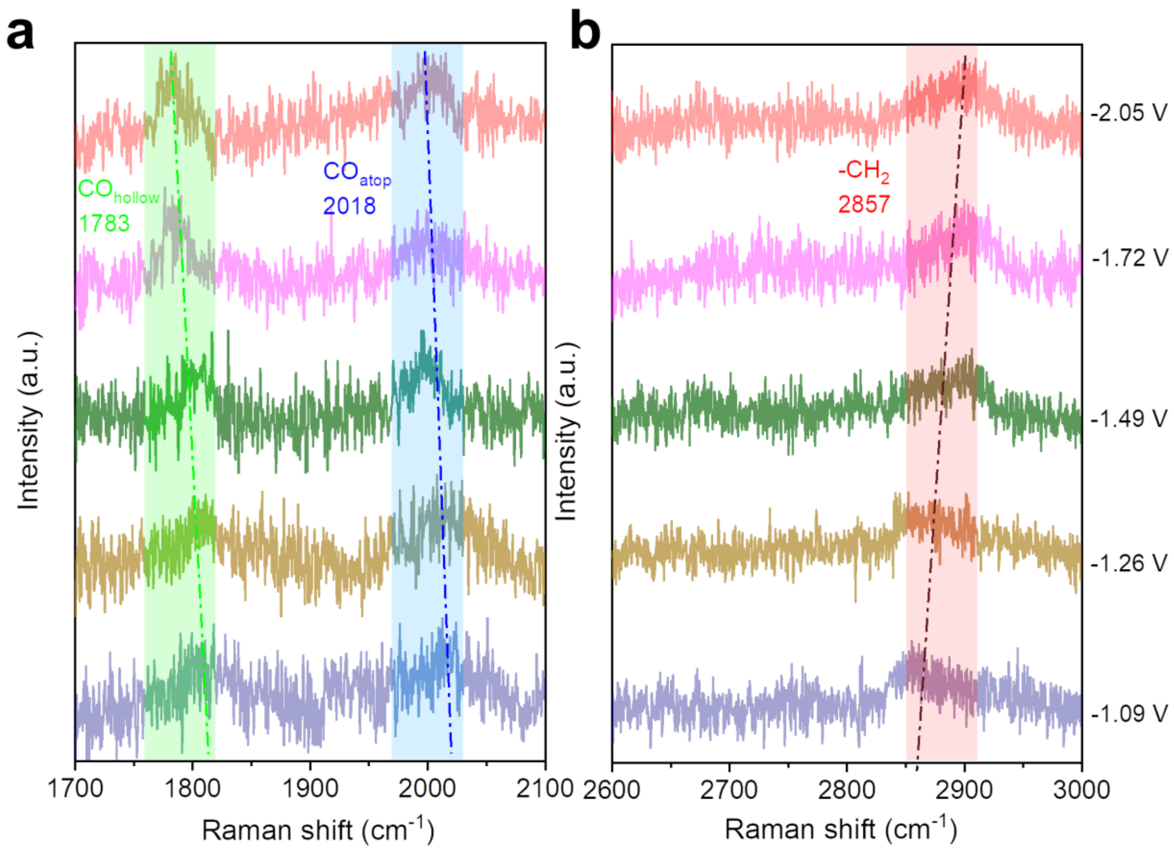

Figure S29. *In-situ* Raman spectroscopy at 1,600-2,100 cm^{-1} (a) and 2,600-3,000 cm^{-1} (b) of the Cu PTFE catalyst measured in 0.05 M H_2SO_4 + 0.5 M K_2SO_4 (acidic) electrolyte.

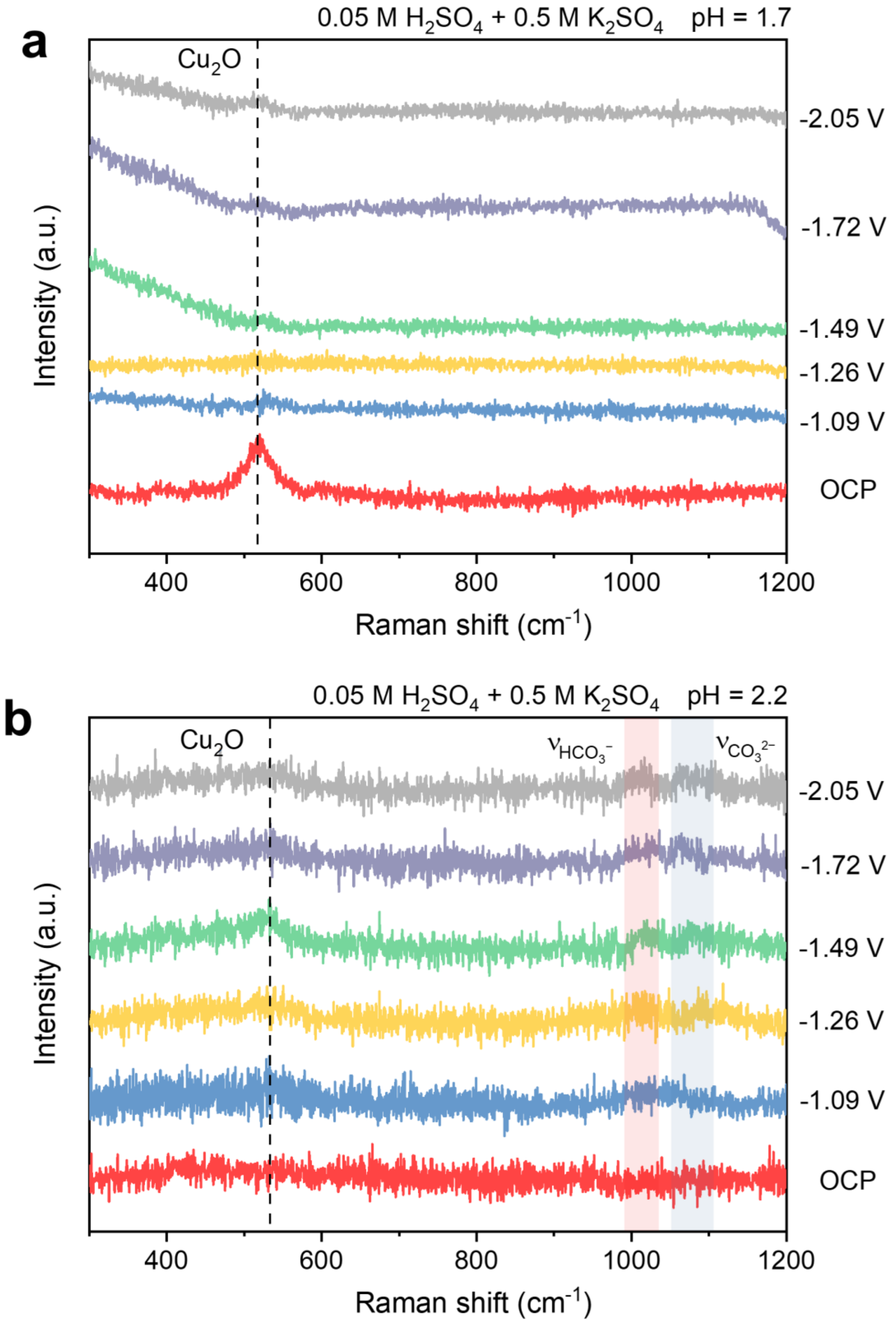

Figure S30. *In-situ* Raman spectroscopy at bulk pH values of: (a) 1.7 and (b) 2.2. HCO_3^- and CO_3^{2-} peaks are observed at 1012 and 1064 cm^{-1}. Voltages are reported vs Ag/AgCl. OCP stands for open-circuit potential, where no current is passing through the system.

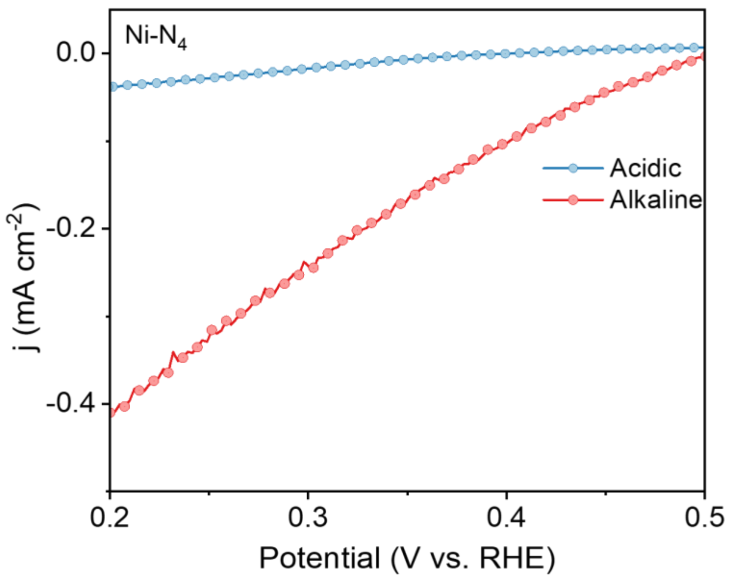

Figure S31. LSV curves for ORR on Ni-N$_4$ in 1 M KOH and in 0.05 M H_2SO_4 with 0.5 M K_2SO_4. For these experiments, pure O_2 was flowed into the gas chamber and electrolyte of the electrochemical flow cell.

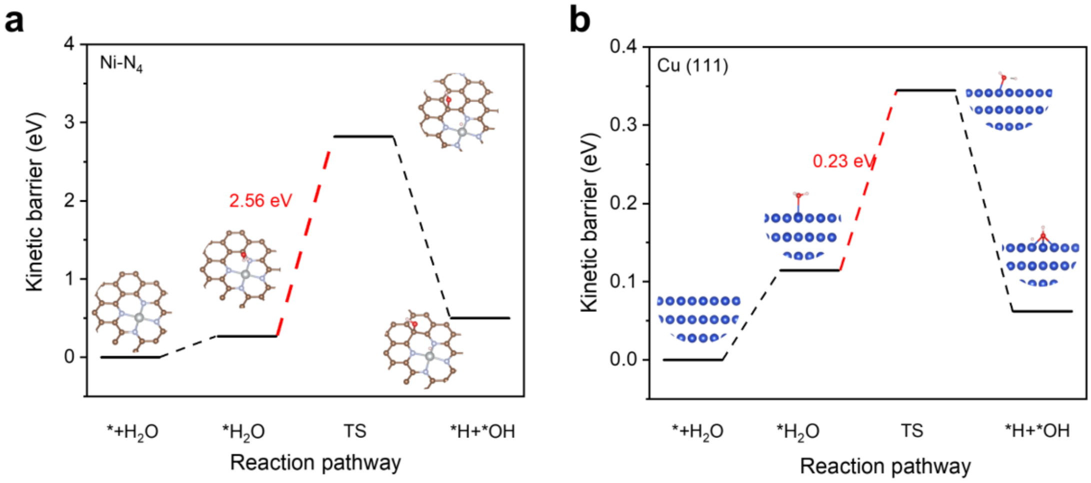

Figure S32. Alkaline HER free energy diagram of (a) Ni-N_4 and (b) Cu (111). The red dotted line represents the activation energy barrier for the rate-determining step.

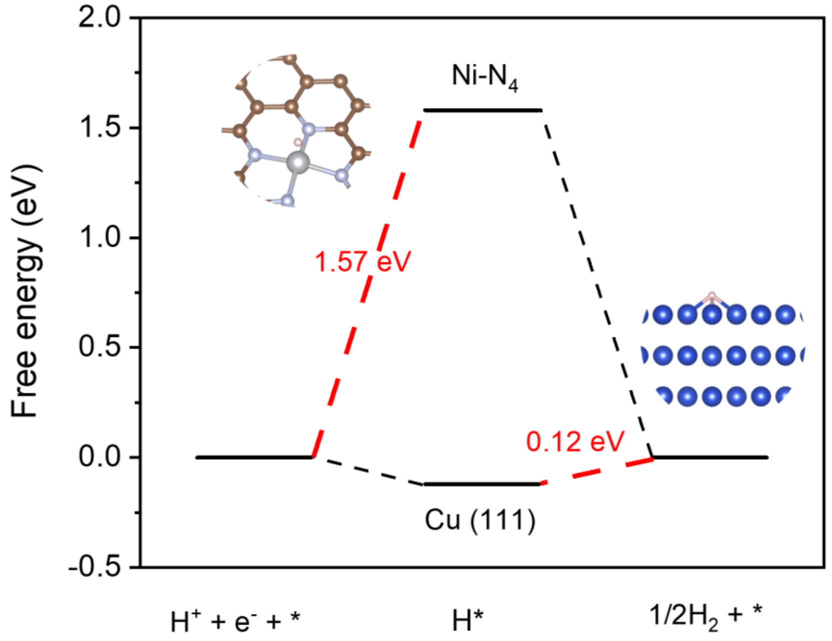

Figure S33. Reaction pathway for proton reduction to hydrogen on Ni-N_4 and Cu (111), involving *H as the intermediate. The red dotted line represents the free energy change for the rate-determining step.

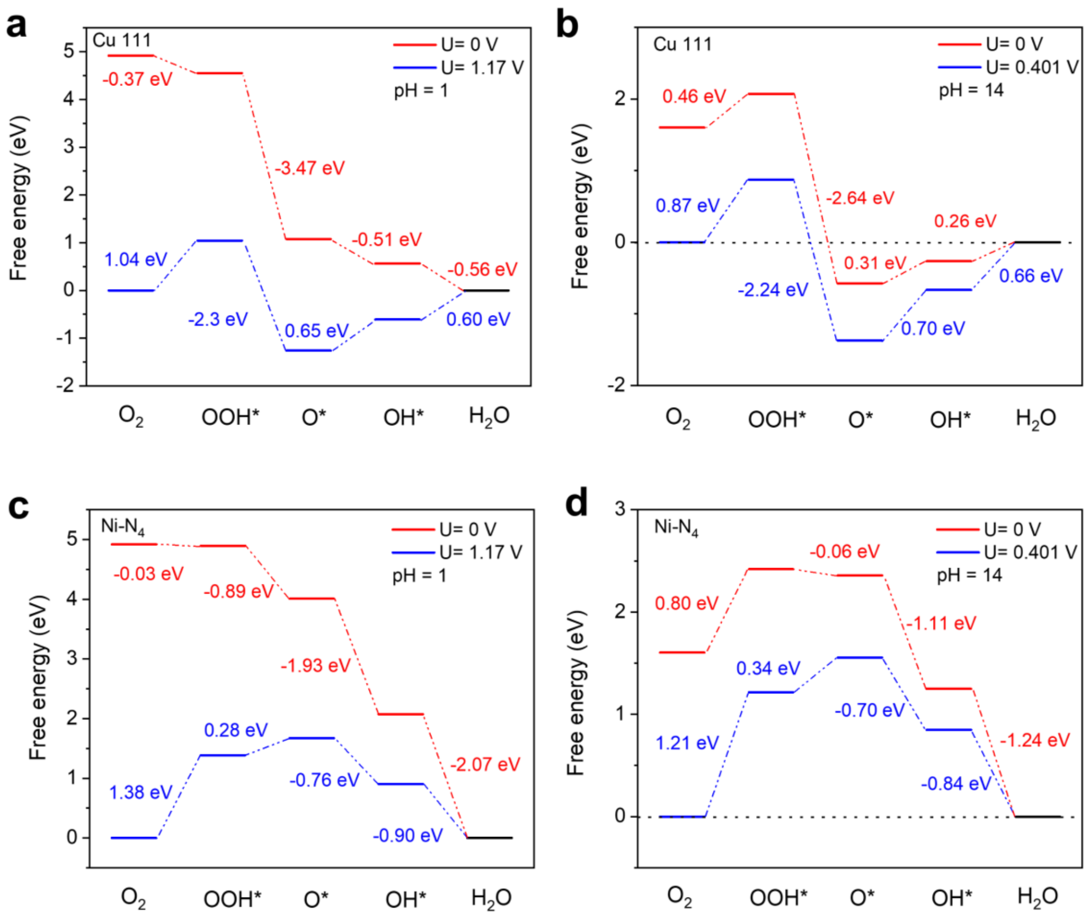

Figure S34. Mechanistic study of the oxygen reduction reaction on Cu (111) and Ni-N_4. Free energy diagrams of the oxygen reduction reaction (ORR) on Cu (111) (a) and (c) Ni-N_4 at 1.17 and 0 V (vs. SHE) in acidic media (pH=1). Free energy diagrams of ORR on Cu (111) (b) and Ni-N_4 (d) at 0.401 and 0 V (vs. SHE) in alkaline media (pH=14) [1].

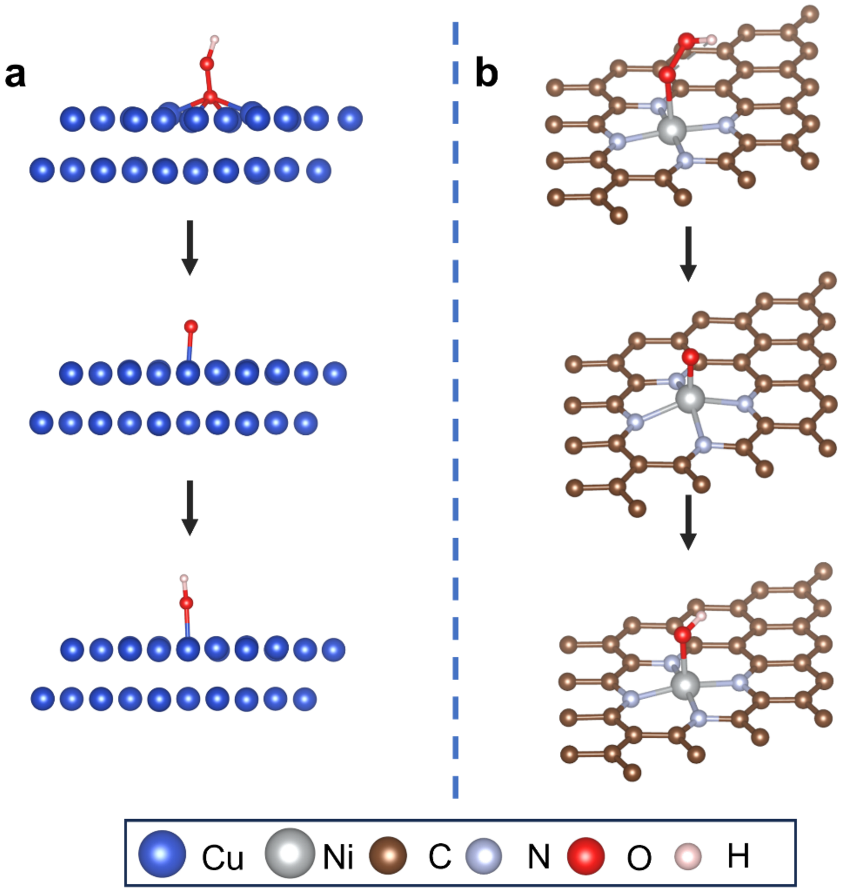

Figure S35. (a) The optimized atomic configurations of ORR intermediate on Cu (111), corresponding to the free energy diagrams in Figure S34a and S34b. (b) The optimized atomic configuration of ORR intermediate states for Ni-N_4, corresponding to the free energy diagrams in Figure S34c and S34d.

The four-electron ORR pathway could be summarized by the following elementary steps [2,3] :

In acidic:

$$O_2(g) + 4H^+ + 4e^- + * \rightarrow OOH* + 3H^+ + 3e^- \quad (1)$$

$$OOH* + 3H^+ + 3e^- \rightarrow O* + 2H^+ + H_2O(l) + 2e^- \quad (2)$$

$$O* + 2H^+ + H_2O(l) + 2e^- \rightarrow OH* + H_2O(l) + H^+ + e^- \quad (3)$$

$$OH* + H_2O(l) + H^+ + e^- \rightarrow 2H_2O(l) + * \quad (4)$$

In alkaline:

$O_2(g) + 2H_2O(l) + 4e^- + * \rightarrow OOH* + OH^- + H_2O(l) +3e^-$ (5)

$OOH* + OH^- + H_2O(l) + 3e^- \rightarrow O* + 2OH^- + H_2O(l) +2e^-$ (6)

$O* + 2OH^- +H_2O(l) + 2e^- \rightarrow OH* + 3OH^- + e^-$ (7)

$OH* + 3OH^- + e^- \rightarrow 4OH^- + *$ (8)

The free energies of the reactants and each intermediate state at an applied electrode potential of U were calculated as follows: $G(U) = \Delta E + \Delta ZPE - T\Delta S - n*eU - n*0.0592 *pH$, where n is the electron number of such state and ΔE represents the change in enthalpy, which is considered from the DFT total energy value, ΔZPE represents the change in zero-point energy and ΔS represents the change in entropy. Since it is difficult to obtain the exact free energy of OOH, O, and OH radicals in the electrolyte solution, the adsorption free energy ΔG_{OOH*}, ΔG_{O*}, and ΔG_{OH*} are used in the calculations.

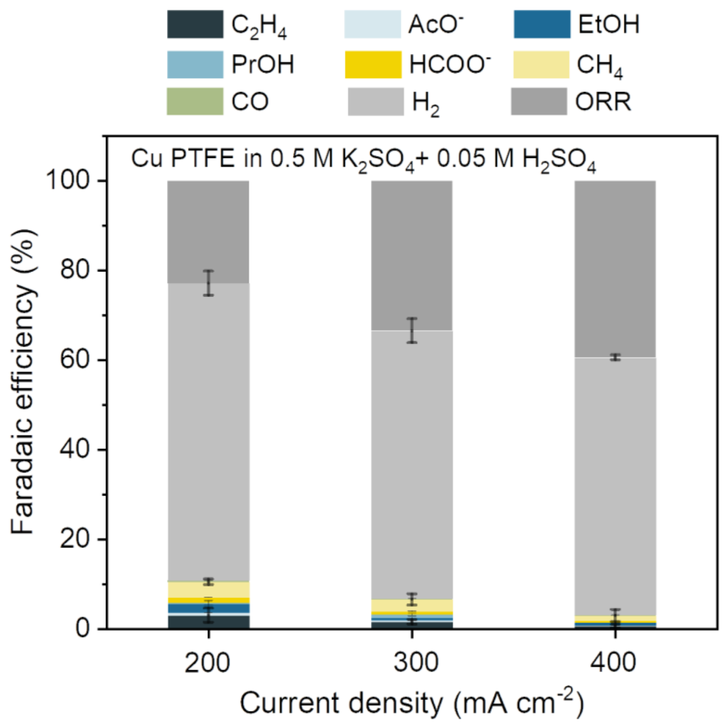

Figure S36. FE results of Cu PTFE in 0.5 M K_2SO_4 + 0.05 M H_2SO_4 at different current densities with simulated flue gas. All the error bars represent standard deviation based on three independent samples.

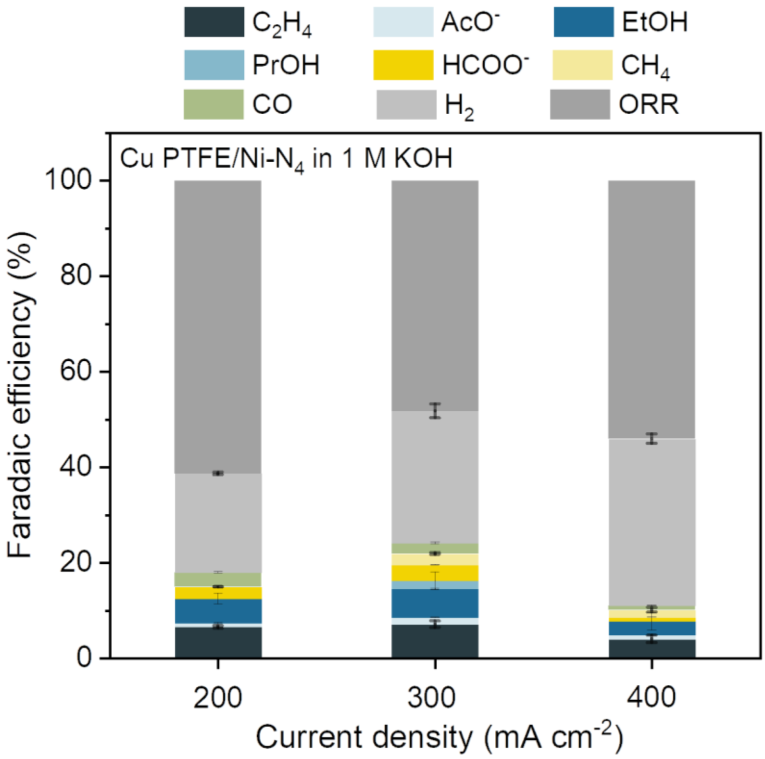

Figure S37. FE results of Cu PTFE/Ni-N_4 in 1 M KOH at different current densities with simulated flue gas. All the error bars represent standard deviation based on three independent samples.

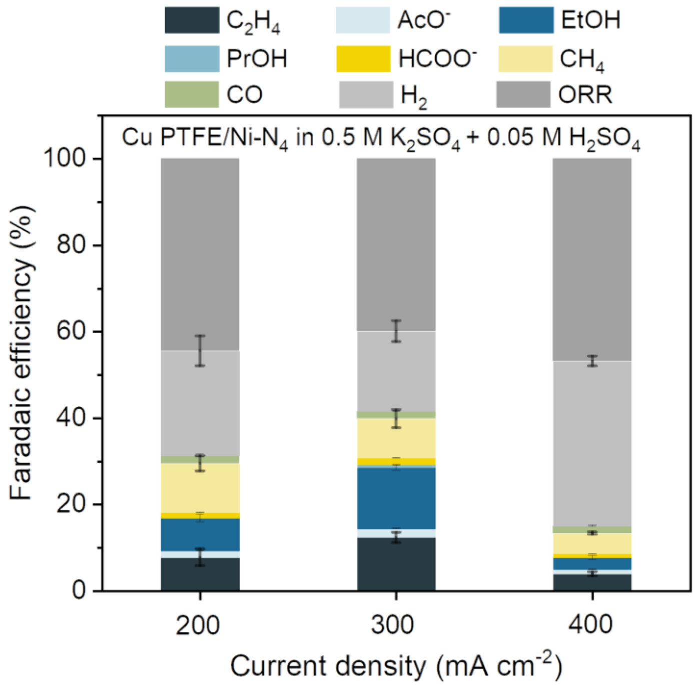

Figure S38. FE results of Cu PTFE/Ni-N_4 in 0.5 M K_2SO_4 + 0.05 M H_2SO_4 at different current densities with simulated flue gas. All the error bars represent standard deviation based on three independent samples.

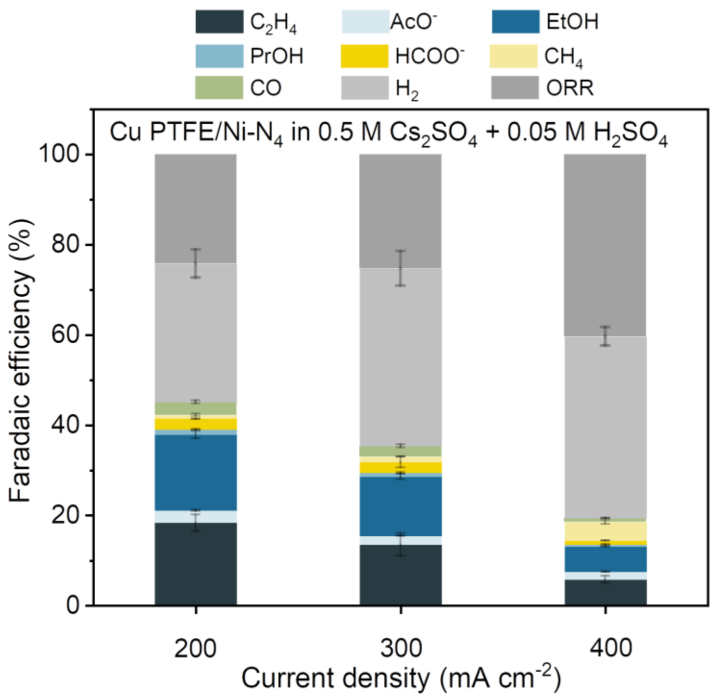

Figure S39. FE results of Cu PTFE/Ni-N4 in 0.05 M H_2SO_4 + 0.5 M Cs_2SO_4 at different current densities with simulated flue gas. All the error bars represent standard deviation based on three independent samples.

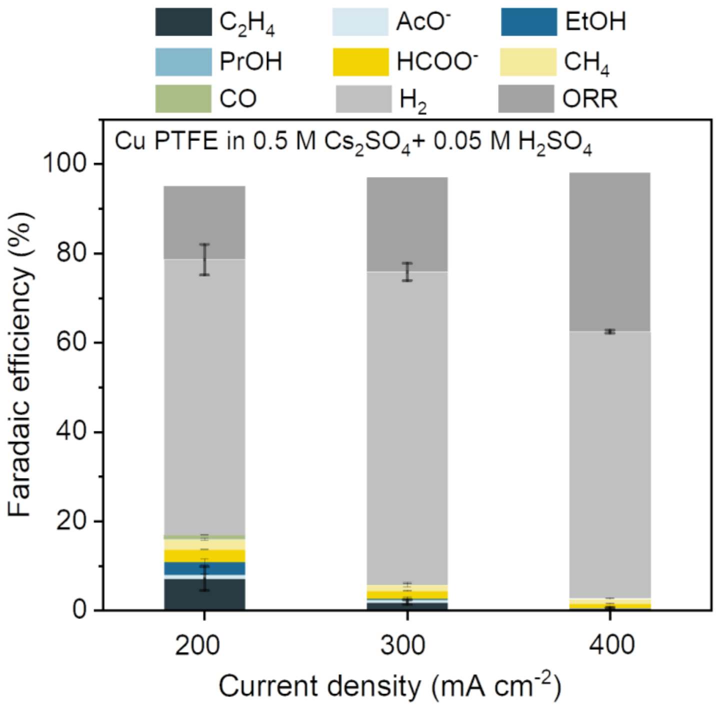

Figure S40. FE results of Cu PTFE in 0.05 M H_2SO_4 + 0.5 M Cs_2SO_4 at different current densities with simulated flue gas. All the error bars represent standard deviation based on three independent samples.

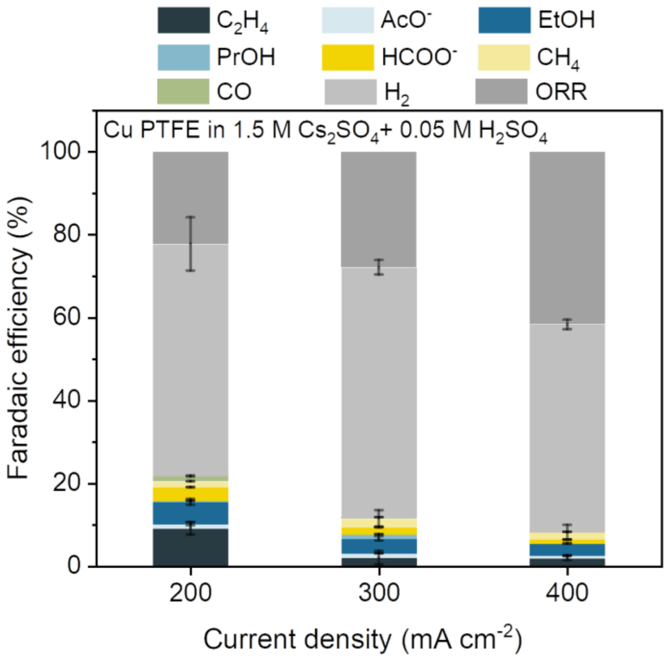

Figure S41. FE results of Cu PTFE in 0.05 M H_2SO_4 + 1.5 M Cs_2SO_4 at different current densities with simulated flue gas. All the error bars represent standard deviation based on three independent samples.

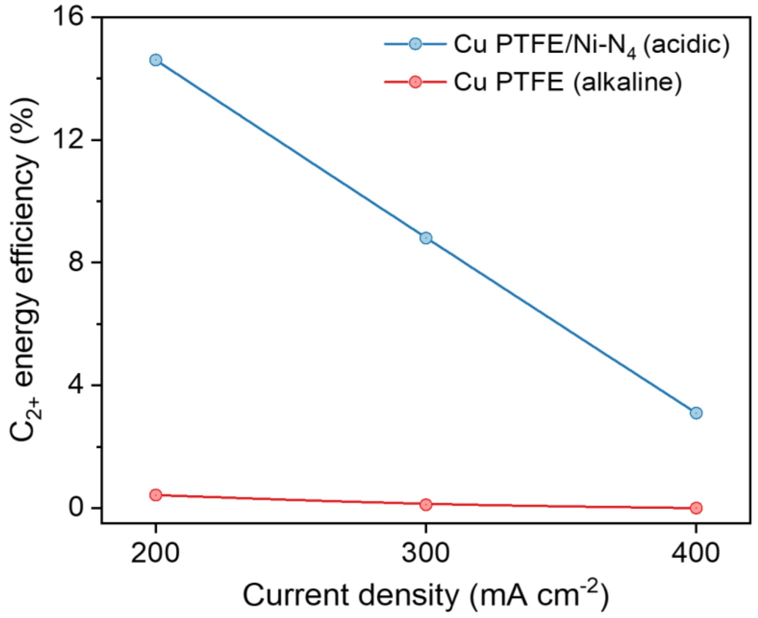

Figure S42. C_{2+} product EE as a function of current density for bare Cu PTFE in alkaline electrolyte (1 M KOH) and Cu PTFE/Ni-N_4 in acidic electrolyte (0.05 M H_2SO_4 + 1.5 M Cs_2SO_4).

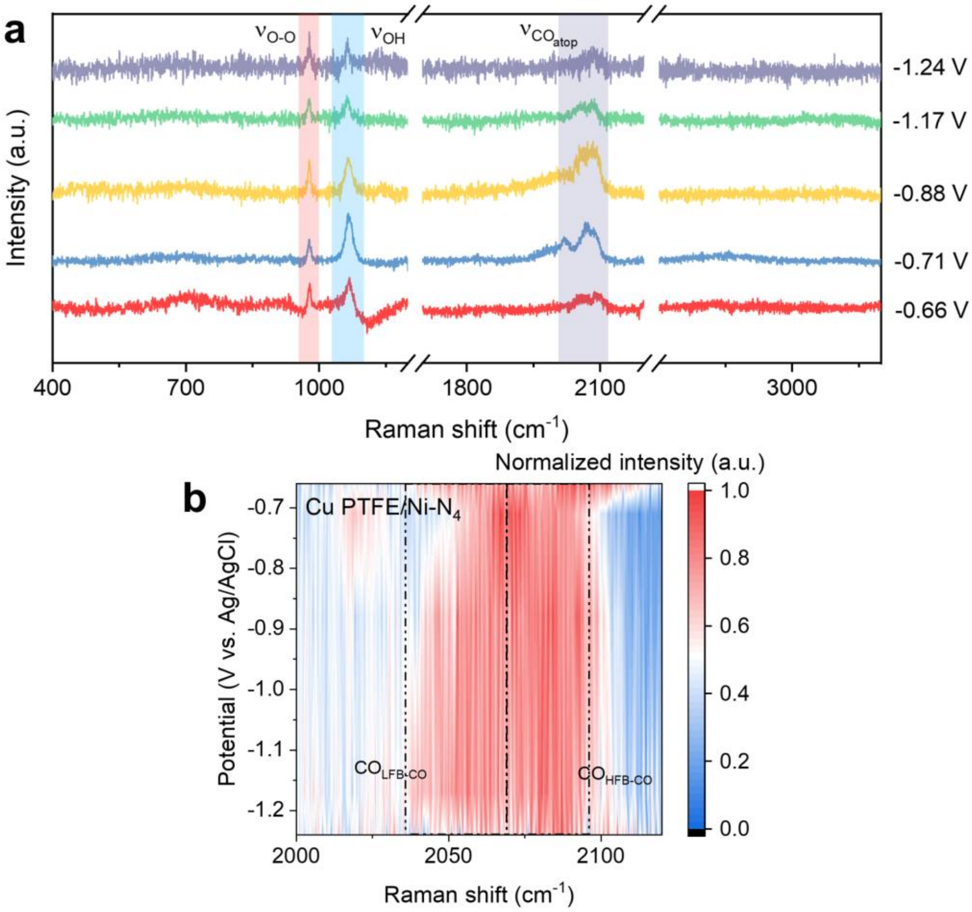

Figure S43. (a) Potential resolved *in-situ* Raman spectroscopy of Cu PTFE/Ni-N_4 in 0.05 M H_2SO_4 + 1.5 M Cs_2SO_4 with simulated flue gas. (b) Raman heatmap of Cu PTFE/Ni-N_4 in the CO region (2000–2120 cm^{-1}), showing the dynamic behavior of adsorbed CO.

Table S1. Faradaic efficiency data for Cu PTFE catalyst in acidic electrolyte at 100, 200, 400 and 600 mA cm^{-2} with pure CO_2 gas. Gas phase products H_2, CO, CH_4 and C_2H_4 are shown here.

**Current density (mA cm^{-2})**	**H_2 (%)**	**Stdev (%)**	**CO (%)**	**Stdev (%)**	**CH_4 (%)**	**Stdev (%)**	**C_2H_4 (%)**	**Stdev (%)**
100	14.2	0.5	5.1	0.3	14.2	1.1	26.9	0.4
200	34.8	1.0	0.5	0.1	20.8	2.2	19.0	0.8
400	53.9	2.0	0.8	0.5	16.1	1.2	9.2	1.2
600	59.2	2.3	0.5	0.4	13.6	1.9	7.9	0.8

Table S2. Faradaic efficiency data for Cu PTFE catalyst in acidic electrolyte at 100, 200, 400 and 600 mA cm^{-2} with pure CO_2 gas. Liquid phase products $HCOO^-$, CH_3COO^-, C_2H_5OH and C_3H_7OH are shown here.

**Current density (mA cm^{-2})**	**$HCOO^-$ (%)**	**Stdev (%)**	**CH_3COO^- (%)**	**Stdev (%)**	**C_2H_5OH (%)**	**Stdev (%)**	**C_3H_7OH (%)**	**Stdev (%)**
100	10.8	0.2	4.1	0.8	18.9	0.1	3.1	0.5
200	9.0	0.6	2.4	0.7	6.5	0.5	1.5	0.6
400	9.3	0.8	1.8	0.6	3.8	0.1	2.4	0.7
600	5.4	1.4	2.8	0.5	4.0	0.2	3.0	0.7

Table S3. Faradaic efficiency data for Ni-N_4 catalyst in acidic electrolyte at 100, 200, 300 and 400 mA cm^{-2} with pure CO_2 gas. Gas phase products H_2, CO, CH_4 and C_2H_4 are shown here.

**Current density (mA cm^{-2})**	**H_2 (%)**	**Stdev (%)**	**CO (%)**	**Stdev (%)**	**CH_4 (%)**	**Stdev (%)**	**C_2H_4 (%)**	**Stdev (%)**
100	10.0	2.8	91.8	2.7	0	0	0	0
200	3.6	0.4	100.4	1.4	0	0	0	0
300	1.7	0.2	103.7	3.5	0	0	0	0
400	1.4	0	105.4	2.1	0	0	0	0

Table S4. Faradaic efficiency data for Cu PTFE/Ni-N_4 catalyst in acidic electrolyte at 100, 200, 400 and 600 mA cm^{-2} with pure CO_2 gas. Gas phase products H_2, CO, CH_4 and C_2H_4 are shown here.

**Current density (mA cm^{-2})**	**H_2 (%)**	**Stdev (%)**	**CO (%)**	**Stdev (%)**	**CH_4 (%)**	**Stdev (%)**	**C_2H_4 (%)**	**Stdev (%)**
100	6.8	0.3	50.8	1.1	0.1	0	14.6	0.8
200	5.6	2.3	24.2	5.5	1.4	0.2	32.6	2.8
400	5.2	0.1	08.1	0.1	2.3	2.0	44.2	0.3
600	7.8	0.5	10.4	0.6	1.1	0.4	42.4	1.7

Table S5. Faradaic efficiency data for Cu PTFE/Ni-N_4 catalyst in acidic electrolyte at 100, 200, 400 and 600 mA cm^{-2} with pure CO_2 gas. Liquid phase products $HCOO^-$, CH_3COO^-, C_2H_5OH and C_3H_7OH are shown here.

**Current density (mA cm^{-2})**	**$HCOO^-$ (%)**	**Stdev (%)**	**CH_3COO^- (%)**	**Stdev (%)**	**C_2H_5OH (%)**	**Stdev (%)**	**C_3H_7OH (%)**	**Stdev (%)**
100	19.6	2.5	0.8	0.4	6.6	0.35	3.2	0.4
200	7.5	2.3	5.3	1.8	25.3	2.5	3.3	1.3
400	2.5	0.8	6.3	2.0	29.6	2.6	2.2	0.2
600	4.7	2.4	5.5	3.8	27.6	1.4	1.1	0.4

Table S6. Potential vs Ag/AgCl for CO_2R in 0.05 M H_2SO_4+0.5 M K_2SO_4 of Cu PTFE and Cu PTFE/Ni-N_4 at applied current density with pure CO_2 gas.

	Cu PTFE	Cu PTFE/Ni-N_4
**100 mA cm^{-2}**	-2.9 V	-2.0 V
**200 mA cm^{-2}**	-3.5 V	-2.2 V
**400 mA cm^{-2}**	-5.3 V	-2.5 V
**600 mA cm^{-2}**	-6.8 V	-3.1 V

Table S7. Full-cell voltage for CO_2R in 0.05 M H_2SO_4+0.5 M K_2SO_4 of Cu PTFE and Cu PTFE/Ni-N_4 at applied current density with pure CO_2 gas.

	Cu PTFE	Cu PTFE/Ni-N_4
**100 mA cm^{-2}**	4.1 V	3.7 V
**200 mA cm^{-2}**	5.3 V	4.4 V
**400 mA cm^{-2}**	7.6 V	6.5 V
**600 mA cm^{-2}**	9.7 V	7.2 V

Table S8. Faradaic efficiency data for Cu PTFE/Ni-N_4 (2 nm Cu) and Cu PTFE/Ni-N_4 (10 nm Cu) catalysts in acidic electrolyte at 200 mA cm^{-2} with pure CO_2 gas. Gas phase products H_2, CO, CH_4 and C_2H_4 are shown here.

Catalysts	H_2 (%)	Stdev (%)	CO (%)	Stdev (%)	CH_4 (%)	Stdev (%)	C_2H_4 (%)	Stdev (%)
Cu PTFE/Ni-N_4 (2 nm Cu)	6.0	/	41.3	/	0.2	/	25.0	/
Cu PTFE/Ni-N_4 (10 nm Cu)	9.2	/	23.7	/	3	/	29.4	/

Table S9. Faradaic efficiency data for Cu PTFE/Ni-N_4 (2 nm Cu) and Cu PTFE/Ni-N_4 (10 nm Cu) catalysts in acidic electrolyte at 200 mA cm^{-2} with pure CO_2 gas. Liquid phase products $HCOO^-$, CH_3COO^-, C_2H_5OH and C_3H_7OH are shown here.

Catalysts	$HCOO^-$ (%)	Stdev (%)	CH_3COO^- (%)	Stdev (%)	C_2H_5OH (%)	Stdev (%)	C_3H_7OH (%)	Stdev (%)
Cu PTFE/Ni-N_4-(2 nm Cu)	7.9	/	2.0	/	18.3	/	1.5	/
Cu PTFE/Ni-N_4(10 nm Cu)	10.0	/	1.7	/	22.9	/	1.7	/

Table S10. Faradaic efficiency data for Cu PTFE/Ni-N_4-1, Cu PTFE/Ni-N_4-2 and Cu PTFE/Ni-N_4-4 catalysts in acidic electrolyte at 200 mA cm^{-2} with pure CO_2 gas. Gas phase products H_2, CO, CH_4 and C_2H_4 are shown here.

Catalysts	H_2 (%)	Stdev (%)	CO (%)	Stdev (%)	CH_4 (%)	Stdev (%)	C_2H_4 (%)	Stdev (%)
Cu PTFE/Ni-N_4-1	7	/	26.5	/	6.5	/	30.6	/
Cu PTFE/Ni-N_4-2	7.5	/	21.4	/	0.6	/	34	/
Cu PTFE/Ni-N_4-4	6.3	/	26.2	/	0.4	/	32.1	/

Table S11. Faradaic efficiency data for Cu PTFE/Ni-N_4-1, Cu PTFE/Ni-N_4-2 and Cu PTFE/Ni-N_4-4 catalysts in acidic electrolyte at 200 mA cm^{-2} with pure CO_2 gas. Liquid phase products $HCOO^-$, CH_3COO^-, C_2H_5OH and C_3H_7OH are shown here.

Catalysts	$HCOO^-$ (%)	Stdev (%)	CH_3COO^- (%)	Stdev (%)	C_2H_5OH (%)	Stdev (%)	C_3H_7OH (%)	Stdev (%)
Cu PTFE/Ni-N_4-1	7.8	/	4.99	/	15.2	/	1.48	/
Cu PTFE/Ni-N_4-2	3.3	/	6.93	/	28.05	/	2.0	/
Cu PTFE/Ni-N_4-4	7.7	/	3.3	/	23.9	/	2.0	/

Table S12. The ratio between the intensities of the bands of Cu PTFE/Ni-N_4 was summarized in Table S12**.**

Potential (V vs. Ag/AgCl)	$^*CO_{(hollow + bridge)}$	$^*CO_{atop}$	$^*CO_{(hollow + bridge)}$ /$^*CO_{atop}$
-1.09	15.9	0	1:0
-1.26	15.7	4.57	0.8:0.2
-1.49	18.7	8.2	0.7:0.3
-1.72	9.9	8.5	0.54:0.45
-2.05	0	17.4	0:1

Table S13. The ratio between the intensities of the bands of Cu PTFE was summarized in Table S13**.**

Potential (V vs. Ag/AgCl)	$^*CO_{(hollow + bridge)}$	$^*CO_{atop}$	$^*CO_{(hollow + bridge\ )}$/$^*CO_{atop}$
-1.09	15.8	23.3	0.4:0.6
-1.26	12.0	17.6	0.4:0.6
-1.49	11.7	12.37	0.5:0.5
-1.72	15.8	13.9	0.53:0.47
-2.05	13.8	11.7	0.54:0.46

The analysis method for the *in-situ* Raman spectroscopy results in this paper is based on prior literature [4-6]. Specifically, each spectral band was deconvoluted into individual bands for $^*CO_{atop}$, $^*CO_{hollow}$ and $^*CO_{bridge}$ using Lorentzian fitting.

Table S14. Faradaic efficiency data for Cu PTFE catalyst in 1 M KOH at 200, 300 and 400 mA cm^{-2} with flue gas. ORR and gas phase products (H_2, CO, CH_4 and C_2H_4) are shown here.

Current density (mA cm^{-2})	ORR (%)	H_2 (%)	Stdev (%)	CO (%)	Stdev (%)	CH_4 (%)	Stdev (%)	C_2H_4 (%)	Stdev (%)
200	40.5	56.4	0.3	0.1	0	0.2	0.2	1.8	1.0
300	43.3	54.8	0.3	0	0	0.1	0.1	1.3	0
400	54.0	44.8	0.2	0	0	0.2	0.2	0.6	0

Table S15. Faradaic efficiency data for Cu PTFE catalyst 1 M KOH at 200, 300 and 400 mA cm^{-2} with flue gas. Liquid phase products $HCOO^-$, CH_3COO^-, C_2H_5OH and C_3H_7OH are shown here.

Current density (mA cm^{-2})	$HCOO^-$ (%)	Stdev (%)	CH_3COO^- (%)	Stdev (%)	C_2H_5OH (%)	Stdev (%)	C_3H_7OH (%)	Stdev (%)
200	0.2	0.4	0.5	0	/	/	/	/
300	/	/	0.2	0	/	/	/	/
400	/	/	0.1	0	/	/	/	/

Table S16. Faradaic efficiency data for Cu PTFE catalyst in 0.05 M H_2SO_4+0.5 M K_2SO_4 at 200, 300 and 400 mA cm^{-2} with flue gas. ORR and gas phase products (H_2, CO, CH_4 and C_2H_4) are shown here.

**Current density (mA cm^{-2})**	**ORR (%)**	**H_2 (%)**	**Stdev (%)**	**CO (%)**	**Stdev (%)**	**CH_4 (%)**	**Stdev (%)**	**C_2H_4 (%)**	**Stdev (%)**
200	22.8	66.3	2.6	0.3	0	3.5	0.1	3.0	1.5
300	26.6	59.7	2.9	0.2	0	2.6	0.1	1.5	0.5
400	39.4	57.5	0.5	0.1	0	1.3	0.1	0.5	0.1

Table S17. Faradaic efficiency data for Cu PTFE catalyst in 0.05 M H_2SO_4+0.5 M K_2SO_4 at 200, 300 and 400 mA cm^{-2} with flue gas. Liquid phase products $HCOO^-$, CH_3COO^-, C_2H_5OH and C_3H_7OH are shown here.

**Current density (mA cm^{-2})**	**$HCOO^-$ (%)**	**Stdev (%)**	**CH_3COO^- (%)**	**Stdev (%)**	**C_2H_5OH (%)**	**Stdev (%)**	**C_3H_7OH (%)**	**Stdev (%)**
200	1.1	0	0.5	0	1.9	0.7	0.3	0
300	0.7	0	0.3	0.1	0.5	0.1	0.7	0
400	0.3	0	0.1	0	0.5	0	0	0

Table S18. Faradaic efficiency data for Cu PTFE/Ni-N_4 catalyst in 1 M KOH at 200, 300 and 400 mA cm^{-2} with flue gas. ORR and gas phase products (H_2, CO, CH_4 and C_2H_4) are shown here.

**Current density (mA cm^{-2})**	**ORR (%)**	**H_2 (%)**	**Stdev (%)**	**CO (%)**	**Stdev (%)**	**CH_4 (%)**	**Stdev (%)**	**C_2H_4 (%)**	**Stdev (%)**
200	61.3	20.6	0.2	3.0	0.1	0	0	6.5	0.2
300	48.2	27.6	1.4	2.2	0.1	2.3	0.1	7.1	0.6
400	54.0	34.9	0.9	0.9	0	1.5	0.4	4.0	0.7

Table S19. Faradaic efficiency data for Cu PTFE/Ni-N_4 catalyst 1 M KOH at 200, 300 and 400 mA cm^{-2} with flue gas. Liquid phase products $HCOO^-$, CH_3COO^-, C_2H_5OH and C_3H_7OH are shown here.

**Current density (mA cm^{-2})**	**$HCOO^-$ (%)**	**Stdev (%)**	**CH_3COO^- (%)**	**Stdev (%)**	**C_2H_5OH (%)**	**Stdev (%)**	**C_3H_7OH (%)**	**Stdev (%)**
200	2.4	0.1	0.7	0.1	5.1	1.1	0	0
300	3.3	0	1.4	0	5.9	0	1.7	1.8
400	0.8	0	0.7	0	2.9	1.8	0	0

Table S20. Faradaic efficiency data for Cu PTFE/Ni-N_4 catalyst in 0.05 M H_2SO_4+0.5 M K_2SO_4 at 200, 300 and 400 mA cm^{-2} with flue gas. ORR and gas phase products (H_2, CO, CH_4 and C_2H_4) are shown here.

Current density (mA cm^{-2})	ORR (%)	H_2 (%)	Stdev (%)	CO (%)	Stdev (%)	CH_4 (%)	Stdev (%)	C_2H_4 (%)	Stdev (%)
200	44.4	24.2	3.4	1.8	0.3	11.4	1.7	7.6	1.9
300	39.9	18.4	2.4	1.7	0	9.0	2.1	12.2	1.2
400	46.8	38.1	1.1	1.7	0.1	4.7	0.2	3.8	0.4

Table S21. Faradaic efficiency data for Cu PTFE/Ni-N_4 catalyst 0.05 M H_2SO_4+0.5 M K_2SO_4 at 200, 300 and 400 mA cm^{-2} with flue gas. Liquid phase products $HCOO^-$, CH_3COO^-, C_2H_5OH and C_3H_7OH are shown here.

Current density (mA cm^{-2})	$HCOO^-$ (%)	Stdev (%)	CH_3COO^- (%)	Stdev (%)	C_2H_5OH (%)	Stdev (%)	C_3H_7OH (%)	Stdev (%)
200	1.2	0	1.5	0	7.6	0.8	0	0
300	1.6	0	2.0	0.1	14.1	0.5	0.6	0
400	0.8	0	1.0	0	2.7	0.4	0	0

Table S22. Potential vs Ag/AgCl for CO_2R in 1 M KOH of Cu PTFE and Cu PTFE/Ni-N_4 at applied current density with flue gas.

	Cu PTFE	Cu PTFE/Ni-N_4
**200 mA cm^{-2}**	-2.7 V	-2.3 V
**300 mA cm^{-2}**	-3.0 V	-2.6 V
**400 mA cm^{-2}**	-3.3 V	-2.9 V

Table S23. Potential vs Ag/AgCl for CO_2R in 0.05 M H_2SO_4+0.5 M K_2SO_4 of Cu PTFE and Cu PTFE/Ni-N_4 at applied current density with flue gas.

	Cu PTFE	Cu PTFE/Ni-N_4
**200 mA cm^{-2}**	-3.2 V	- 2.2 V
**300 mA cm^{-2}**	-3.9 V	-2.5 V
**400 mA cm^{-2}**	-4.7 V	-3.1 V

Table S24. Full-cell voltage for CO_2R in 1 M KOH of Cu PTFE at applied current density with flue gas.

	Cu PTFE
**200 mA cm^{-2}**	6.0 V
**300 mA cm^{-2}**	8.0 V
**400 mA cm^{-2}**	9.7 V

Table 25. Faradaic efficiency data for Cu PTFE/Ni-N_4 catalyst in 0.05 M H_2SO_4+0.5 M Cs_2SO_4 at 200, 300 and 400 mA cm^{-2} with flue gas. ORR and gas phase products (H_2, CO, CH_4 and C_2H_4) are shown here.

Current density (mA cm^{-2})	ORR (%)	H_2 (%)	Stdev (%)	CO (%)	Stdev (%)	CH_4 (%)	Stdev (%)	C_2H_4 (%)	Stdev (%)
200	24.1	30.6	3.1	2.8	0.3	0.8	0.2	18.3	1.8
300	25.2	39.3	3.8	2.3	0.3	1.2	0	13.5	2.5
400	40.3	40.2	2.0	0.8	0	4.1	0.4	5.7	0.7

Table S26. Faradaic efficiency data for Cu PTFE/Ni-N_4 catalyst 0.05 M H_2SO_4+0.5 M Cs_2SO_4 at 200, 300 and 400 mA cm^{-2} with flue gas. Liquid phase products $HCOO^-$, CH_3COO^-, C_2H_5OH and C_3H_7OH are shown here.

Current density (mA cm^{-2})	$HCOO^-$ (%)	Stdev (%)	CH_3COO^- (%)	Stdev (%)	C_2H_5OH (%)	Stdev (%)	C_3H_7OH (%)	Stdev (%)
200	2.3	0	2.7	0.1	16.8	0.8	1.1	0
300	2.3	1.1	1.9	0	13.1	0.6	0.8	0.1
400	0.8	0	1.6	0	5.6	0.1	0.4	0

Table S27. Potential vs Ag/AgCl for CO_2R in 0.05 M H_2SO_4+0.5 M Cs_2SO_4 of and Cu PTFE/Ni-N_4 at applied current density with flue gas.

	Cu PTFE/Ni-N_4
**200 mA cm^{-2}**	-2.3 V
**300 mA cm^{-2}**	-2.9 V
**400 mA cm^{-2}**	-3.2 V

Table S28. Full-cell voltage for CO_2R in 0.05 M H_2SO_4+0.5 M Cs_2SO_4 of Cu PTFE/Ni-N_4 at applied current density with flue gas.

	Cu PTFE/Ni-N_4
**200 mA cm^{-2}**	4.5 V
**300 mA cm^{-2}**	5.4 V
**400 mA cm^{-2}**	6.4 V

Table S29. Faradaic efficiency data for Cu PTFE catalyst in 0.05 M H_2SO_4+0.5 M Cs_2SO_4 at 200, 300 and 400 mA cm^{-2} with flue gas. ORR and gas phase products (H_2, CO, CH_4 and C_2H_4) are shown here.

Current density (mA cm^{-2})	ORR (%)	H_2 (%)	Stdev (%)	CO (%)	Stdev (%)	CH_4 (%)	Stdev (%)	C_2H_4 (%)	Stdev (%)
200	16.4	61.6	3.4	1.0	0.2	2.2	0.2	7.0	2.6
300	21.2	69.8	1.9	0.2	0.1	1.3	0.5	1.7	0.5
400	35.5	59.6	0.3	0.1	0.3	1.1	0.1	0.3	0.1

Table S30. Faradaic efficiency data for Cu PTFE catalyst H_2SO_4+0.5 M Cs_2SO_4 at 200, 300 and 400 mA cm^{-2} with flue gas. Liquid phase products $HCOO^-$, CH_3COO^-, C_2H_5OH and C_3H_7OH are shown here.

Current density (mA cm^{-2})	$HCOO^-$ (%)	Stdev (%)	CH_3COO^- (%)	Stdev (%)	C_2H_5OH (%)	Stdev (%)	C_3H_7OH (%)	Stdev (%)
200	2.7	0.8	0.9	0.1	2.7	0.7	0	0
300	1.6	0.6	0.5	0.1	0.3	0.2	0	0
400	0.8	0.1	0.2	0.1	0	0	0	0

Table S31. Potential vs Ag/AgCl for CO_2R in 0.05 M H_2SO_4+0.5 M Cs_2SO_4 of and Cu PTFE at applied current density with flue gas.

	Cu PTFE
**200 mA cm^{-2}**	2.7 V
**300 mA cm^{-2}**	3.2 V
**400 mA cm^{-2}**	4.1 V

Table S32. Full-cell voltage for CO_2R in 0.05 M H_2SO_4+0.5 M Cs_2SO_4 of Cu PTFE at applied current density with flue gas.

	Cu PTFE
**200 mA cm^{-2}**	4.6
**300 mA cm^{-2}**	5.7
**400 mA cm^{-2}**	6.6

Table S33. Faradaic efficiency data for Cu PTFE/Ni-N_4 catalyst in 0.05 M H_2SO_4+1.5 M Cs_2SO_4 at 200, 300 and 400 mA cm^{-2} with flue gas. ORR and gas phase products (H_2, CO, CH_4 and C_2H_4) are shown here.

Current density (mA cm^{-2})	ORR (%)	H_2 (%)	Stdev (%)	CO (%)	Stdev (%)	CH_4 (%)	Stdev (%)	C_2H_4 (%)	Stdev (%)
200	21.0	25.6	3.1	1.8	0.3	0.5	0.4	21.4	0.2
300	31.4	26.3	3.8	1.3	0.03	1.5	0	15.3	4.0
400	45.8	34.8	2.0	0.8	0	3.4	0.2	6.0	0.4

Table S34. Faradaic efficiency data for Cu PTFE/Ni-N_4 catalyst H_2SO_4+1.5 M Cs_2SO_4 at 200, 300 and 400 mA cm^{-2} with flue gas. Liquid phase products $HCOO^-$, CH_3COO^-, C_2H_5OH and C_3H_7OH are shown here.

Current density (mA cm^{-2})	$HCOO^-$ (%)	Stdev (%)	CH_3COO^- (%)	Stdev (%)	C_2H_5OH (%)	Stdev (%)	C_3H_7OH (%)	Stdev (%)
200	4.3	0	3.0	0.5	20.0	2.3	1.8	0
300	5.0	1.3	1.9	0	15.1	0.2	1.8	0
400	0.5	0	1.6	0	6.0	1.5	0.6	0

Table S35. Potential vs Ag/AgCl for CO_2R in 0.05 M H_2SO_4+1.5 M Cs_2SO_4 of and Cu PTFE/Ni-N_4 at applied current density with flue gas.

	Cu PTFE/Ni-N_4
**200 mA cm^{-2}**	-2.0 V
**300 mA cm^{-2}**	-2.4 V
**400 mA cm^{-2}**	-2.7 V

Table S36. Full-cell voltage for CO_2R in 0.05 M H_2SO_4+1.5 M Cs_2SO_4 of Cu PTFE/Ni-N_4 at applied current density with flue gas.

	Cu PTFE/Ni-N_4
**200 mA cm^{-2}**	3.7 V
**300 mA cm^{-2}**	4.5 V
**400 mA cm^{-2}**	5.5 V

Table S37. Faradaic efficiency data for Cu PTFE catalyst in 0.05 M H_2SO_4+1.5 M Cs_2SO_4 at 200, 300 and 400 mA cm^{-2} with flue gas. ORR and gas phase products (H_2, CO, CH_4 and C_2H_4) are shown here.

Current density (mA cm^{-2})	ORR (%)	H_2 (%)	Stdev (%)	CO (%)	Stdev (%)	CH_4 (%)	Stdev (%)	C_2H_4 (%)	Stdev (%)
200	22.2	56.0	6.4	1.2	0.1	1.4	0	9.1	1.4
300	27.8	60.3	1.7	0.2	0	2.1	1.9	2.0	1.6
400	41.6	50.0	1.1	0.1	0	1.6	1.7	1.9	0.5

Table S38. Faradaic efficiency data for Cu PTFE catalyst H_2SO_4+1.5 M Cs_2SO_4 at 200, 300 and 400 mA cm^{-2} with flue gas. Liquid phase products $HCOO^-$, CH_3COO^-, C_2H_5OH and C_3H_7OH are shown here.

Current density (mA cm^{-2})	$HCOO^-$ (%)	Stdev (%)	CH_3COO^- (%)	Stdev (%)	C_2H_5OH (%)	Stdev (%)	C_3H_7OH (%)	Stdev (%)
200	3.2	0.5	1.0	0.4	5.2	0.7	0.3	0
300	1.7	0.2	1.0	0	3.6	0.4	0.9	0
400	1.0	0.1	0.6	0	2.8	0	0	0

Table S39. Potential vs Ag/AgCl for CO_2R in 0.05 M H_2SO_4+1.5 M Cs_2SO_4 of and Cu PTFE at applied current density with flue gas.

	Cu PTFE
**200 mA cm^{-2}**	2.1 V
**300 mA cm^{-2}**	2.4 V
**400 mA cm^{-2}**	3.4 V

Table S40. Full-cell voltage for CO_2R in 0.05 M H_2SO_4+1.5 M Cs_2SO_4 of Cu PTFE at applied current density with flue gas.

	Cu PTFE
**200 mA cm^{-2}**	4.0 V
**300 mA cm^{-2}**	4.7 V
**400 mA cm^{-2}**	5.8 V

Table S41. C_{2+} FE for CO_2R with simulated flue gas in 0.05 M H_2SO_4 + 1.5 M Cs_2SO_4 using Cu PTFE/Ni-N_4. The average full-cell voltage over the 24 h period is 3.7 V.

Time	**1 h**	**4 h**	**7 h**	**10 h**	**13 h**
C_{2+} FE	46.8%	45.1%	44.2%	46.3%	43.2%
Time	**16 h**	**19 h**	**22 h**	**24 h**	
C_{2+} FE	41.6%	40.5%	0.4	38.4%	

Supplementary References

1. Lv, Q.; Si, W.; He, J.; Sun, L.; Zhang, C.; Wang, N.; Yang, Z.; Li, X.; Wang, X.; Deng, W.; Long, Y.; Huang, C.; Li, Y., Selectively nitrogen-doped carbon materials as superior metal-free catalysts for oxygen reduction. *Nature Communications* 2018, 9 (1), 3376.

2. Zheng, Y.; Jiao, Y.; Zhu, Y.; Cai, Q.; Vasileff, A.; Li, L. H.; Han, Y.; Chen, Y.; Qiao, S.-Z., Molecule-Level g-C_3N_4 Coordinated Transition Metals as a New Class of Electrocatalysts for Oxygen Electrode Reactions. *Journal of the American Chemical Society* 2017, 139 (9), 3336-3339.

3. Jiao, Y.; Zheng, Y.; Jaroniec, M.; Qiao, S. Z., Origin of the Electrocatalytic Oxygen Reduction Activity of Graphene-Based Catalysts: A Roadmap to Achieve the Best Performance. *Journal of the American Chemical Society* 2014, 136 (11), 4394-4403.

4. Li, F., Thevenon, A., Rosas-Hernández, A. et al. Molecular tuning of CO_2-to-ethylene conversion. *Nature* 2020, 577, 509–513.

5. Wei, P., Gao, D., Liu, T. et al. Coverage-driven selectivity switch from ethylene to acetate in high-rate CO_2/CO electrolysis. *Nature Nanotechnology* 2023, 18, 299–306.

6. Qi, K., Zhang, Y., Onofrio, N. et al. Unlocking direct CO_2 electrolysis to C_3 products via electrolyte supersaturation. *Nature Catalysis* 2023, 6, 319–331.
